# Supplementary material for: Cost-effectiveness of a primary healthcare intervention to treat male lower urinary tract symptoms: the TRIUMPH cluster randomised controlled trial
Source: BMJ Open. 2024 Jan 30;14(1):e075704. doi: 10.1136/bmjopen-2023-075704 (PMC10828877; doi:10.1136/bmjopen-2023-075704)
Supplement: Supplementary data [file bmjopen-2023-075704supp001.pdf]

Table 1. Output for multilevel model to estimate adjusted mean differences in costs (n=866)

Total Costs	Coefficient	Bootstrap (SE)	P-value	95% Confident Intervals
Intervention arm	-29.99	32.93	0.36	-94.52 to 34.55
Centre	-54.12	29.36	0.07	-111.66 to 3.43
GP IT System	-56.73	38.20	0.14	-131.59 to 18.14
Practice size	0.00	0.00	0.31	-0.00 to 0.00
IMD	5.68	3.13	0.07	-0.45 to 11.82
Constant	235.56	52.52	0.00	132.62 to 338.50
ICC	0.05	0.02		0.02 to 0.11

SE: Standard Error; IMD: Index of Multiple Deprivation; GP (General Practice) Informatic System; ICC: Intraclass correlation.

Table 2. Output for multilevel model to estimate adjusted mean differences in QALYs (n=866)

Total QALYs	Coefficient	Bootstrap (SE)	P-value	95% CI
Intervention arm	0.00	0.01	0.90	-0.01 to 0.01
Centre	-0.00	0.01	0.60	-0.02 to 0.01
Practice size	1.96e-07	2.75e-07	0.47	-3.42e-07 to 7.35e-07
IMD	-0.00	0.00	0.32	-0.00 to 0.00
Baseline utility	0.81	0.018	0.00	0.78 to 0.85
Constant	0.16	0.019	0.00	0.13 to 0.20
ICC	6.48e-35	9.76e-19		0 to 1

QALYs: Quality Adjusted Life Years; CI: Confidence Intervals; IMD: Indices of Multiple Deprivation; ICC: Intraclass correlation.

Table 3. Additional sensitivity and subgroup analyses

		Adjusted*, mean (95% CI)**		Incremental adjusted mean (95% CI **)		
Trial arm	n	Costs (£)	QALYs	Costs (£)	QALYs	INMB (£) at £20,000/QALY (95% CI)
Complete Case Analysis with MLM: Adjusting for 12-month pre-consent GP consultation visits						
Intervention	413	£277.53 (£245.93 to £309.14)	0.84 (0.83 to 0.84)	-£3.02 (-£49.52 to £44.02)	0.001 (-0.011 to 0.014)	£21.50 (-£246.93 to £289.03)
Usual care	453	£280.56 (£244.16 to £316.96)	0.84 (0.83 to 0.84)			
Complete Case Analysis with MLM: Subgroup analysis for participants completing 12-month follow up before 11th March 2020						
Intervention	236	£255.12 (£133.88 to £376.35)	0.83 (0.81 to 0.84)	-£33.04 (-£209.94 to £438.39)	-0.001 (-0.027 to 0.010)	£48.01 (-£225.83 to £321.85)
Usual care	169	£288.16 (£23.20 to £553.11)	0.84 (0.82 to 0.85)			
Complete Case Analysis with MLM: Subgroup analysis for participants completing 12-month follow up from 11th March 2020						
Intervention	177	£229.31 (£119.77 to £338.85)	0.86 (0.81 to 0.90)	-£67.77 (-£219.86 to £74.92)	0.029 (0.002 to 0.133)	£35.54 (-£182.20 to £253.28)
Usual care	284	£297.07 (£204.54 to £389.61)	0.83 (0.76 to 0.90)			

*Adjusted for centre, practice-size and area-level deprivation. In addition, costs were adjusted for general practice IT system and QALYs were adjusted for baseline utility.**Bootstrapped bias corrected and accelerated confidence intervals; SUR= Seemingly Unrelated Regression; INMB= Incremental Net Monetary Benefit; MLM= Multilevel model.
