# Supplementary material for: Cost-effectiveness of a primary healthcare intervention to treat male lower urinary tract symptoms: the TRIUMPH cluster randomised controlled trial
Source: BMJ Open. 2024 Jan 30;14(1):e075704. doi: 10.1136/bmjopen-2023-075704 (PMC10828877; doi:10.1136/bmjopen-2023-075704)
Supplement: Author's manuscript [file bmjopen-2023-075704.draft_revisions.pdf]

BMJ Open

BMJ Open is committed to open peer review. As part of this commitment we make the peer review history of every article we publish publicly available.

When an article is published we post the peer reviewers' comments and the authors' responses online. We also post the versions of the paper that were used during peer review. These are the versions that the peer review comments apply to.

The versions of the paper that follow are the versions that were submitted during the peer review process. They are not the versions of record or the final published versions. They should not be cited or distributed as the published version of this manuscript.

BMJ Open is an open access journal and the full, final, typeset and author-corrected version of record of the manuscript is available on our site with no access controls, subscription charges or pay-per-view fees (http://bmjopen.bmj.com).

If you have any questions on BMJ Open's open peer review process please email info.bmjopen@bmj.com

BMJ Open

# Cost-effectiveness of a primary healthcare intervention to treat male lower urinary tract symptoms: the TRIUMPH cluster randomised controlled trial

| | |
|---|---|
| Journal: | *BMJ Open* |
| Manuscript ID | bmjopen-2023-075704 |
| Article Type: | Original research |
| Date Submitted by the Author: | 17-May-2023 |
| Complete List of Authors: | Cochrane, Madeleine; University of Bristol, Population Health Sciences<br>Drake, Marcus J.; Imperial College London, Department of Surgery & Cancer<br>Worthington, Jo; University of Bristol, Population Health Sciences<br>Frost, Jessica; University of Bristol, Population Health Sciences<br>Cotterill, Nikki; University of the West of England, Department of Nursing & Midwifery<br>Fader, Mandy; University of Southampton, School of Health Sciences<br>McGeagh, Lucy ; Oxford Brookes University, Faculty of Health and Life Sciences<br>Hashim, Hashim; North Bristol NHS Trust Southmead Hospital, Bristol Urological Institute<br>Lane, Athene; University of Bristol, Population Health Sciences<br>Macaulay, Margaret; University of Southampton, Faculty of Health Sciences<br>MacNeill , Stephanie; University of Bristol, Population Health Sciences<br>Rees, Jonathan; Brockway Medical Centre<br>Ridd, Matthew; University of Bristol, Population Health Sciences<br>Robles, Luke; University of Bristol, NIHR Bristol Biomedical Research Centre<br>Sanderson, Emily; University of Bristol, Population Health Sciences<br>Taylor, Gordon; Public and Patient Involvement Representative<br>Taylor, Jodi; University of Bristol, Population Health Sciences<br>Noble, Sian; University of Bristol, Population Health Sciences |
| Keywords: | HEALTH ECONOMICS, UROLOGY, Primary Health Care |
| | |

SCHOLARONE™
Manuscripts

*I, the Submitting Author has the right to grant and does grant on behalf of all authors of the Work (as defined in the below author licence), an exclusive licence and/or a non-exclusive licence for contributions from authors who are: i) UK Crown employees; ii) where BMJ has agreed a CC-BY licence shall apply, and/or iii) in accordance with the terms applicable for US Federal Government officers or employees acting as part of their official duties; on a worldwide, perpetual, irrevocable, royalty-free basis to BMJ Publishing Group Ltd ("BMJ") its licensees and where the relevant Journal is co-owned by BMJ to the co-owners of the Journal, to publish the Work in this journal and any other BMJ products and to exploit all rights, as set out in our licence.*

*The Submitting Author accepts and understands that any supply made under these terms is made by BMJ to the Submitting Author unless you are acting as an employee on behalf of your employer or a postgraduate student of an affiliated institution which is paying any applicable article publishing charge ("APC") for Open Access articles. Where the Submitting Author wishes to make the Work available on an Open Access basis (and intends to pay the relevant APC), the terms of reuse of such Open Access shall be governed by a Creative Commons licence – details of these licences and which Creative Commons licence will apply to this Work are set out in our licence referred to above.*

*Other than as permitted in any relevant BMJ Author's Self Archiving Policies, I confirm this Work has not been accepted for publication elsewhere, is not being considered for publication elsewhere and does not duplicate material already published. I confirm all authors consent to publication of this Work and authorise the granting of this licence.*

For peer review only

**Cost-effectiveness of a primary healthcare intervention to treat male lower urinary tract symptoms: the TRIUMPH cluster randomised controlled trial**

Madeleine Cochrane* (0000-0003-1856-3293), Marcus J. Drake (0000-0002-6230-2552), Jo Worthington (0000-0002-2860-3511), Jessica Frost (0000-0002-2454-311X), Nikki Cotterill (0000-0001-6921-2712), Mandy Fader (0000-0002-0801-543X), Lucy McGeagh (0000-0002-9226-5115), Hashim Hashim (0000-0003-2467-407X), J Athene Lane (0000-0002-7578-4925), Margaret Macaulay (0000-0003-1737-4589), Stephanie J MacNeill (0000-0001-6553-1433), Jonathan Rees (0000-0001-8014-4407), Matthew J Ridd (0000-0002-7954-8823), Luke A Robles (0000-0003-2882-9868), Emily Sanderson (0000-0003-2268-4194), Gordon Taylor (0000-0002-8029-2503), Jodi Taylor (0000-0001-7171-8923), Sian Noble (0000-0002-8011-0722)

Population Health Sciences, Bristol Medical School, University of Bristol, 1-5 Whiteladies Road, Bristol, BS8 1NU, UK. Madeleine Cochrane, Senior Research Associate in Health Economics, J Athene Lane, Professor of Trials Research, Jo Worthington, Senior Trial Manager, Jessica Frost, Trial Manager, Stephanie J MacNeill, Senior Lecturer in Medical Statistics, Emily Sanderson, Research Associate in Medical Statistics, Jodi Taylor, Head of Trial Management and Operations (Bristol Trials Centre), Sian Noble, Senior Lecturer in Health Economics

Faculty of Medicine, Department of Surgery & Cancer, Imperial College London, London W12 0HS, UK. Marcus J. Drake, Chair in Neurological Urology

Department of Nursing & Midwifery, Faculty of Health & Applied Sciences, University of the West of England, Bristol, UK. Nikki Cotterill, Associate Professor in Continence Care

School of Health Sciences, University of Southampton, University Road, Southampton, SO17 1BJ, UK. Mandy Fader, Professor of Continence Technology, Margaret Macaulay, Senior Research Nurse

Bristol Urological Institute, Level 3, Learning and Research Building, North Bristol NHS Trust, Southmead Hospital, Bristol, BS10 5NB, UK. Hashim Hashim, Professor, Consultant Urological Surgeon

NIHR Bristol Biomedical Research Centre, University Hospitals Bristol and Weston NHS Foundation Trust and the University of Bristol, Level 3, University Hospitals Bristol Education Centre, Upper Maudlin Street, Bristol, BS2 8AE, UK. Luke A Robles, Senior Research Associate in Cancer

Oxford Institute Nursing, Midwifery and Allied Health Research, Oxford Brookes University, OX3 0BP. Lucy McGeagh, Senior Research Fellow

Tyntesfield Medical Group, Brockway Medical Centre, 8 Brockway, Nailsea, Bristol, BS48 1BZ, UK Jonathan Rees, General Practitioner Partner

Public and patient involvement representative, UK. Gordon Taylor, PPI representative



For peer review only - http://bmjopen.bmj.com/site/about/guidelines.xhtml

Centre of Academic Primary Care, Population Health Sciences, Bristol Medical School, University of Bristol, Canynge Hall, 39 Whatley Road, Bristol, BS8 2PS, UK. Matthew J Ridd, Professor of Primary Health Care

*Correspondence to: Dr Madeleine Cochrane: ORCiD ID 0000-0003-1856-3293; madeleine.cochrane@bristol.ac.uk; Bristol Trials Centre, Population Health Sciences, Bristol Medical School, University of Bristol, Canynge Hall, 39 Whatley Road, Bristol, BS8 2PS, UK

## Transparency statement

Cochrane had full access to all the data in the study and affirms that the manuscript is an honest, accurate, and transparent account of the study being reported; that no important aspects of the study have been omitted; and that any discrepancies from the study as originally planned (and, if relevant, registered) have been explained.

## Contributor and guarantor information

Cochrane attests that all listed authors meet authorship criteria and that no others meeting the criteria have been omitted.

*Conception or design:* MC, SN, MDJ, JAL, NC, MF, LM, HH, SM, MJR, ES, JR, LAR and GT assisted with the study design. JW and JF managed the coordination of the study. MM and JT also assisted with the coordination of the study.

*Analysis:* MC and SN conducted the analysis for the economic evaluation.

*Drafting the manuscript:* MC and SN developed the manuscript for the economic evaluation. All authors contributed to the oversight of the study via the TMG, read and commented on manuscript drafts and approved the final manuscript.

*Obtaining funding:* MJD was the Chief Investigator of the TRIUMPH study. The study was funded by National Institute for Health Research (NIHR), Health Technology Assessment (HTA) programme, funding number 16/90/03. The views expressed in this publication are those of the authors and not necessarily those of the NHS, the NIHR or the Department of Health and Social Care. MJR is partially funded by a Post-Doctoral Research Fellowship from the NIHR (PDF-2014-07-013).

## Copyright/license for publication

The Corresponding Author has the right to grant on behalf of all authors and does grant on behalf of all authors, a worldwide licence to the Publishers and its licensees in perpetuity, in all forms, formats and media (whether known now or created in the future), to i) publish, reproduce, distribute, display



For peer review only - http://bmjopen.bmj.com/site/about/guidelines.xhtml

and store the Contribution, ii) translate the Contribution into other languages, create adaptations, reprints, include within collections and create summaries, extracts and/or, abstracts of the Contribution, iii) create any other derivative work(s) based on the Contribution, iv) to exploit all subsidiary rights in the Contribution, v) the inclusion of electronic links from the Contribution to third party material where-ever it may be located; and, vi) licence any third party to do any or all of the above.

## Competing interests declaration

Prof Marcus Drake reports personal fees from Astellas and Pfizer, outside the submitted work. Dr Jonathan Rees is chair of the Primary Care Urology Society which has received non-promotional sponsorship for annual meetings from Ferring, Astellas, Neotract and IMedicare. He has also received speaker fees from Astellas Pharmaceuticals. Prof Hashim Hashim reports personal fees from Medtronic, Astellas, Allergan and Boston Scientific, outside the submitted work. Prof Athene Lane reports receiving funding for the clinical trials unit (CTU) of which she was co-director, and is currently an active member on the NIHR CTU Standing Advisory Committee. Dr Matthew Ridd has been on several NIHR committees including the Systematic Reviews NIHR Cochrane Incentive Awards, HTA General Committee, Evidence Synthesis Programme Grants Committee, NIHR Incentive Awards Committee and is currently on the Evidence Synthesis Programme Advisory Group. Stephanie MacNeill is an active member of the HTA General Committee.

## Role of funding source

This study was funded by the National Institute for Health Research (NIHR) HTA programme, funding number 16/90/03. The funder had no role in the design and conduct of the study; collection, management, analysis, and interpretation of the data; preparation, review, or approval of the manuscript; and decision to submit the manuscript for publication.

Bristol Trials Centre receives National Institute for Health Research CTU Support Funding. This funding has been awarded to support us in developing and supporting NIHR trials. The views expressed are those of Bristol Trials Centre and not necessarily those of the NIHR or the Department of Health and Social Care.

## Disclaimer

The views expressed in this article are those of the authors and do not necessarily reflect the opinions of the National Health Service, the National Institute for Health Research, or the Department of Health.



For peer review only - http://bmjopen.bmj.com/site/about/guidelines.xhtml

## Ethical approval

Approval from the NRES North West Preston Ethics Committee (18/NW/0135) was received on 11 April 2018 and applied to all NHS sites who took part in the study. All participants provided their written, informed consent to participation before entering the study

## Data sharing/ data availability statement

All data requests should be submitted to the corresponding author for consideration. Access to anonymised data may be granted following review.

## Dissemination

TRIUMPH study participants will be informed of the study results via our study website (bristol.ac.uk/triumph-study). In addition, they will be sent details of the results via the study newsletter.

## Acknowledgements

This study was designed and delivered in collaboration with the Bristol Randomised Trials Collaboration (BRTC), a UKCRC registered clinical trials unit which, as part of the Bristol Trials Centre, is in receipt of National Institute for Health Research CTU support funding. The University of Bristol acted as the Sponsor for this trial and the trial was hosted by the NHS Bristol, North Somerset and South Gloucestershire Clinical Commissioning Group (CCG). The TRIUMPH Research team acknowledges the support of the National Institute for Health Research Clinical Research Network (NIHR CRN). Study data were collected and managed using REDCap hosted at the University of Bristol.

The authors would like to thank all participants, principal investigators and their teams at each of the TRIUMPH study sites for their involvement, and the West of England and Wessex CRNs for their role in the study. The authors would also like to thank the members of the Patient Advisory Group, Trial Steering Committee and Data Monitoring Committee.

## Trial registration

Trial registration was prospective before enrolling the first patient. ISRCTN registry number: ISRCTN11669964. Registration date: 12/04/2018.



For peer review only - http://bmjopen.bmj.com/site/about/guidelines.xhtml

## Abstract

**Objectives:** To estimate the cost-effectiveness of a primary care intervention for male lower urinary tract symptoms (LUTS) compared with usual care.

**Design:** Economic evaluation alongside a cluster randomised controlled trial from an UK National Health Service (NHS) perspective with a 12-month time horizon.

**Setting:** Thirty NHS general practice sites in England.

**Participants:** 1,077 men aged 18 or older identified in primary care with bothersome LUTS.

**Interventions:** A standardised and manualised intervention for the treatment of bothersome LUTS was compared to usual care. The intervention group (n=524) received a standardised information booklet with guidance on conservative treatment for LUTS, urinary symptom assessment, and follow up contacts for 12 weeks. The usual care group (n=553) followed local guidelines between general practice sites.

**Measures:** Resource use was obtained from electronic health records, trial staff and participants, and valued using UK reference costs. Quality-adjusted life years (QALYs) were calculated from the EQ-5D-5L questionnaire. Adjusted mean differences in costs and QALYs and incremental net monetary benefit were estimated.

**Results:** 866 of 1,077 (80.4%) participants had complete data and were included in the base-case analysis. Over the 12-month follow-up period, intervention and usual care arms had similar mean adjusted costs and QALYs. Mean differences were lower in the intervention arm for adjusted costs -£29.99 (95% CI -£109.84 to £22.63) while higher in the intervention arm for adjusted QALYs 0.001 (95% CI -0.011 to 0.014). The incremental net monetary benefit statistic was £48.01 (95% CI -£225.83 to £321.85) at the NICE UK threshold of £20,000 per QALY. The cost-effectiveness acceptability curve showed a 63% probability of the intervention arm being cost-effective at this threshold.

**Conclusions:** Costs and QALYs were similar between the two arms at 12 months follow-up. This indicates that the intervention can be implemented at low cost.

**Trial registration:** ISRCTN11669964.



For peer review only - http://bmjopen.bmj.com/site/about/guidelines.xhtml

## Strengths and limitations of this study

- Our study presents the first economic evaluation carried out alongside a large multi-centre definitive trial to provide evidence on the value for money of providing a manualised and standardised non-pharmacological intervention for the treatment of LUTS in primary care.
- Using electronic health records to capture primary care resource use resulted in high rates of complete cost data which are higher than those reported in the economic evaluation literature.
- Our electronic health records derived from England's two main general practice IT systems. A limitation of this method was that the IT systems provided different levels of detail for some components of the consultation and medication data.

For peer review only



For peer review only - http://bmjopen.bmj.com/site/about/guidelines.xhtml

## Introduction

Lower urinary tract symptoms (LUTS) is a bothersome condition among the ageing male population with a prevalence of up to 30% in men over 65 years old (1). LUTS includes storage, voiding and post-micturition symptoms, and can have a considerable impact on quality of life (2). Causes of LUTS include benign prostate enlargement (BPE), detrusor muscle weakness or overactivity, prostate inflammation and lifestyle habits. Evidence-based national guidelines from the UK's National Institute for Health and Care Excellence (NICE) recommend conservative management interventions (such as fluid and caffeine intake, bladder training, urethral compression and release, and pelvic floor muscle exercises) at initial assessments for LUTS, once serious underlying conditions (e.g. urinary tract infection, prostate cancer, neurological disease) have been excluded (1).

Conservative management options (3), are time-consuming and complex to implement due to the range of possible symptoms and causes of LUTS. Initial evidence from a small single centre randomised controlled trial (RCT) suggests conservative self-management interventions could play a role in the delivery of treatment for LUTS. However, this smaller trial only evaluated effectiveness and not cost-effectiveness (4). The present study, TReatIng Urinary symptoms in Men in Primary Healthcare (TRIUMPH), sought to provide evidence on whether the provision of a manualised and standardised non-pharmacological intervention in primary care, is effective and cost-effective compared to usual care. The effectiveness results, reported elsewhere, showed improved symptoms for men with LUTS and these benefits were sustained for a full year [insert: cross-reference to effectiveness paper]. The current cost-effectiveness study is conducted from an NHS perspective over a 12-month time horizon, with the intention to inform clinical practice decisions within primary care.

## Methods

### Target population and setting

Participants in the TRIUMPH study were men aged 18 or over, who considered themselves to have bothersome LUTS and had presented to primary care within the past five years with at least one symptom of LUTS. The trial was carried out across thirty general practice sites in Southwest England. The National Research Ethics Service North West Preston Ethics Committee (18/NW/0135) gave ethical approval and all study participants gave written, informed consent before participating in the study.

### Comparators

The intervention arm, a manualised and standardised intervention, involved the provision of a information booklet with guidance on conservative interventions for LUTS. Participants had an initial symptom assessment with a Nurse or Healthcare Assistant in order to understand their symptom needs, bothersomeness, and impact on their quality of life, as well as their personal circumstances. This was delivered face-to-face. Participants were then directed to specific sections of the information booklet. Participants were offered up to three further contacts from the healthcare professional. The



For peer review only - http://bmjopen.bmj.com/site/about/guidelines.xhtml

first of the three was offered via telephone while for the remaining two, participants were given the option to receive these via telephone, email or text. The comparator group continued to receive care as usual.

**Study design**

The economic evaluation was conducted alongside the TRIUMPH trial, a multicentre cluster RCT, and evaluated costs and outcomes from an NHS perspective over a 12-month time horizon. Detailed methods for the design of the TRIUMPH trial [ISRCTN11669964] are described elsewhere (5). In brief, thirty general practice sites were randomised on a 1:1 basis to either deliver the intervention or to continue care as usual. Randomisation was minimised by NIHR Clinical Research Network (CRN) centre (West of England and Wessex), practice size and area-level deprivation.

**Patient and public involvement**

A Patient Advisory Group (PAG) was set up to ensure patient and public involvement (PPI) throughout all stages of the research. PPI representatives were involved in the design of the study, ensuring all study material were usable and appropriate for a non-specialist audience. In particular, the PPI group reviewed and advised on the following study components: TRIUMPH intervention booklet, patient-facing questionnaires, newsletters, website, initial qualitative results relating to men's experiences of the patient pathways for LUTS within the NHS, and agreeing to plans for implementing and disseminating the study results. Two PPI representatives also played a significant role in planning and chairing the PAG meetings. In addition, both the Trial Management Group and Trial Steering Committee each included a patient representative who contributed to the management and conduct of the trial.

**Outcomes**

The quality-adjusted life year (QALY) was used as the outcome measure, as recommended in the UK's reference case (6) for interventions funded by the NHS. Health status was measured using the EQ-5D-5L questionnaire and EQ-5D-VAS at baseline, six and 12 months. At baseline the questionnaire was administered via post, while at six and 12 months it could be completed by post or online. Participants' EQ-5D-5L scores were mapped to the EQ-5D-3L valuation set using a validated mapping function by van Hout et al. (7). This enabled a utility score to be calculated for each participant based on published pre-existing utility scores derived from a representative sample of the UK population. Utility scores were then combined with length of life to calculate the QALYs for each participant using an area-under-the-curve approach (8). The approach accounted for any deaths that occurred during the 12-month trial period.

**Resource use data collection and valuation**

Resource use data was obtained for intervention training and delivery costs, primary care consultations and LUTS- related medication and secondary care. Data for each individual patient's 12-month follow up period was captured via the following sources: trial management records, case report forms, primary care Electronic Health Records (EHR) data and self-report questionnaires.



For peer review only - http://bmjopen.bmj.com/site/about/guidelines.xhtml

Intervention-related resources in terms of training, including staff time, travel and teleconference expenses, were obtained primarily through trial management records. Expert opinion from the Lead Nurses was used to capture duration of the training. Resources used in the delivery of the intervention in terms of staff time for the initial clinic visit and subsequent follow-up were prospectively recorded on case report forms.

Primary care EHRs were used to capture: (1) all primary care consultations with a GP, Nurse, Healthcare Assistant or Pharmacist; (2) LUTS-related prescribed medication; and for participants who had a urology referral recorded in their primary care EHRs but had no self-report data (3) LUTS-related secondary care (outpatient, day case, inpatient and accident and emergency (A&E) visits). Database searches were built to extract EHRs from primary care administration systems (EMIS and SystmOne). Records were extracted from each general practice site at least one month after their last recruited participant had reached their 12-month follow up date.

Self-report questionnaires at six and 12-month follow-up, administered either online or via post, were used to capture secondary care LUTS-related healthcare use (outpatient, day case, inpatient and A&E visits). A urology clinician was consulted to identify LUTS-related secondary care visits.

Details of how the resources were valued are reported in Table 1. 2018/19 costs were used to value the resource use. The NHS cost inflation index (NHSCII) was used to inflate a unit cost to current prices if it was unavailable for the year of analysis (9).

**Table 1. Resources collected and their valuation**

| Resources | Unit cost (£) | Source of unit cost |
|---|---|---|
| GP Surgery visit | 34[a,b] | Curtis and Burns, 2019(9) |
| GP Telephone call | 26.27 [a,b,c] | Curtis and Burns, 2019(9) |
| GP Home visit | 87.28 [a,b,d] | Curtis and Burns, 2019(9) |
| Practice Nurse Surgery visit | 10.85 [b,e] | Curtis and Burns, 2019(9) |
| Practice Nurse Telephone call | 8.25 [b,f] | Curtis and Burns, 2019(9) |
| Healthcare Assistant Surgery visit | 6.67[e] | Curtis and Burns, 2019(9) |
| Healthcare Assistant Telephone call | 5.07[f] | Curtis and Burns, 2019(9) |
| Pharmacist Surgery visit | 11.63[e] | Curtis and Burns, 2019(9) |
| Pharmacist Surgery Telephone call | 8.84[f] | Curtis and Burns, 2019(9) |
| NHS 111 call | 12.26 | Pope et al. 2017 (10) |
| Trainers (Professor; Lead Nurses) | Varies | Research Institution's pay scales, 2018/19[g] (11); Curtis and Burns, 2019[h] (9) |
| Intervention booklet | 6.15[i] | Research Institution's printing service charge(12) |
| Intervention visit time per minute | Varies[j] | Curtis and Burns, 2019(9) |
| Follow up contact time per minute | Varies[k] | Curtis and Burns, 2019(9) |
| Teleconference call per minute | 0.15 | BT Conference Call service charge(13) |
| Car mileage | 0.45[l] | HM Revenue and Customs (14) |
| Outpatient procedures | Varies | NHS National Cost Collection, 2018/19(15) |
| Outpatient visits | 110[m] | NHS National Cost Collection, 2018/19(15) |
| Day cases | Varies | NHS National Cost Collection, 2018/19(15) |
| Inpatient admissions | Varies | NHS National Cost Collection, 2018/19(15) |
| Accident & Emergency attendances | 159 | NHS National Cost Collection, 2018/19(15) |
| Medications | Varies[n] | Prescription Cost Analysis, 2019(16); NHS Drug Tariff, 2019(17) |

[a]Excluding direct care staff costs
[b]Including qualifications
[c]Based on the assumption of a 7.1 minute telephone consultation, as reported in earlier unit cost series by Curtis and Burns (18)

[d]Based on the assumption of a 11.4 minute home visit and 12 minutes of travel, as reported in earlier unit cost series by Curtis and Burns (18)
[e]Based on the assumption of a 15.5 minute surgery consultation, as reported for a Practice Nurse in earlier unit cost series by Curtis and Burns. It was assumed the Nurses (practice and research), HCAs and Pharmacists had the same surgery consultation length (18)
[f]There is no published unit cost for non-triage telephone consultations for Practice Nurses, HCAs and Pharmacists. Telephone consultations were assumed to be 11.8 minutes for these healthcare professionals. This was based on data in Curtis and Burns 2015 and 2019 series, which indicates face-to-face and triage telephone consultations delivered by a Practice Nurse are approximately 1.5 times longer than those provided by a GP(9, 18).
[g]Including basic salary, National Insurance and superannuation
[h]Assuming Band 6 for all Lead Nurses
[i] All booklets had to be prepared in advance, it was therefore assumed all participants randomised to the intervention arm incurred the booklet cost
[j]For intervention visits, length of visit was reported in minutes in the case report form. Unit cost varied depending upon which staff type delivered the intervention.
[k]For telephone follow-ups, length of visit was reported in minutes in the case report form. Unit cost varied depending upon which staff type delivered the intervention. For texts and emails, the number of these were reported in the case report form. Time taken to send each text and/or email was assumed to be two minutes.
[l]Distance travelled was calculated by measuring the distance between the staff's workplace and the training locations

[m]For outpatient visits a weighted average of the unit costs for consultant and non-consultant led visits was used.

[n]In the minority of instances where a unit cost was not available in the Prescription Cost Analysis, then the unit costs from the 2019 NHS Drug Tariff were used

**Analysis**

An analysis plan was pre-specified before all data were collected (19). The primary economic analysis was to evaluate costs and outcomes over a 12-month time horizon and so discounting was not required. EHR data were derived from two different IT systems, a standardised cleaning process was therefore implemented to ensure the final analysis dataset was comparable across the two systems. All participants were analysed in accordance with the allocation group they were assigned to at the point of randomisation.

Simple mean imputation was used for a minority of participants who had missing data in relation to their length of follow up visit at week 1 (n=1) and week 4 (n=7) in their case report forms. Additionally, it was assumed zero secondary care visits occurred if other parts of the questionnaire had been completed but the resource use questions had been left blank and the patient had no urology referrals recorded in their EHRs. Total costs were then calculated for each individual participant by summing all the costs across all categories (intervention, primary care, LUTS medications and secondary care) and mean total unadjusted costs per arm were then estimated.

A multilevel model (MLM) was used to account for clustered data and to adjust for the minimisation variables (practice size, IMD and centre) as well as general practice IT system and baseline utility for costs and QALYs (20), respectively. El Alili et al. (21) found MLMs performed better than OLS regression for cluster-based economic evaluations. The MLM was used to calculate the adjusted mean costs and QALYs, and the adjusted mean differences in costs and QALYs between groups. For both costs and QALYs, 95% CIs were estimated using bias-corrected and accelerated (BCa) bootstrapping (21).



For peer review only - http://bmjopen.bmj.com/site/about/guidelines.xhtml

Regression outputs from the MLM facilitated the estimation of an incremental net monetary benefit (INMB) statistic and the associated confidence intervals at NICE's recommended willingness to pay threshold of £20,000 and £30,000 per QALY. A cost-effectiveness acceptability curve (CEAC) was constructed to present the uncertainty over a range of willingness to pay thresholds. This was done using the parametric p-value approach as applied by El Alili et al.(21) and Hoch et al.(4).

One-way sensitivity analyses were conducted to explore methodological uncertainty:

- An alternative analytic model was employed by using a seemingly unrelated regression (SUR) model instead of a MLM.
- Inconsistency in coding the mode of delivery for primary care consultations was explored by re-coding all unit costs for telephone contacts to unit costs for face-to-face contacts.
- Training costs were excluded from the total intervention costs, as it is possible that these costs are captured in the published unit costs.
- Intervention training and delivery costs were excluded.
- The primary analysis model was adjusted for GP consultation costs for the 12-month period pre-consent.
- Missing data assumptions were tested using a range of approaches, including:
  - Multiple imputation by chained equations which use predictive mean matching were combined with the SUR model. The covariates in the multiple imputation model were site ID, baseline utility, centre, practice size, IMD and general practice system. The model was run by allocation group and a randomisation seed was set to ensure reproducible imputations. A total of 25 imputations were performed and combined with Rubin's rule in Stata 16.1 (22).
  - For each arm, zero costs and a QALY value that was 10% higher than the mean QALY value was imputed for participants with missing questionnaire data.
  - For participants with missing questionnaire data, the largest participant cost for secondary care and a QALY value 10% lower than the mean QALY value per arm were imputed.
  - SystmOne EHR based practices did not include data on the quantity of tablets prescribed per medication. In the base-case analysis, EMIS practices data was used to estimate the mean quantity in the SystmOne practices. A sensitivity analysis used the most frequently reported quantity, rather than the mean.

A post-hoc subgroup analysis compared participants who had completed follow-up from 11th March 2020 (where 11th March 2020 reflects when the COVID-19 outbreak was declared internationally as a pandemic) to those who completed follow-up before this date.

**Results**

1,077 men were recruited; either to receive the intervention sites (n=524) or usual care sites (n=553). There was a low rate (5.1%, n=55) of missing EHR data (primary care and medications), and a greater rate of item missingness for the self-report secondary care (12.5%, n=135) and EQ-5D-5L



For peer review only - http://bmjopen.bmj.com/site/about/guidelines.xhtml

(16.1%, n=173) data. This rate of missing data permitted a complete case primary analysis on 866 out of 1,077 (80.4%) participants, 413 (78.8%) and 453 (81.9%) participants in the intervention and usual care arm, respectively.

**Resource use, costs and outcomes**

Table 2 shows that mean resource use and costs from available cases were similar between arms, as were the adjusted mean costs for total primary care consultations, medications and secondary care visits. Mean intervention costs were £39, excluding intervention training costs reduced this to £31. Overall, EQ-5D-5L scores, QALYs and EQ-VAS scores were similar across both groups and all timepoints (Table 3).

Table 2. Resource use and costs from NHS perspective

| Resource category | Intervention (n=524) | | | Usual care (n=553) | | |
|---|---|---|---|---|---|---|
| | n | Resource use, mean (SD) | Cost, mean (SD) (£) | n | Resource use, mean (SD) | Cost, mean (SD) (£) |
| GP consultations all types | 478 | 4.60 (4.67) | 145.86 (162.62) | 544 | 5.00 (5.68) | 158.17 (180.05) |
| Practice Nurse consultations all types | 478 | 2.78 (3.64) | 28.77 (38.58) | 544 | 3.61 (5.75) | 38.41 (58.48) |
| HCA all types of consultations | 478 | 1.16 (2.05) | 7.70 (13.67) | 544 | 0.59 (2.07) | 3.91 (13.82) |
| Pharmacist all types of consultations | 478 | 0.15 (0.54) | 1.60 (5.59) | 544 | 0.15 (0.90) | 1.36 (7.18) |
| Total LUTS medications | 478 | 3.27 (4.74) | 45.77 (100.50) | 544 | 4.29 (5.75) | 42.22 (98.97) |
| NHS 111 encounters | 459 | 0 | 0.00 | 479 | 0.01 (0.14) | 0.08 (1.72) |
| Outpatient visits | 459 | 0.07(0.34) | 8.04(36.75) | 479 | 0.08(0.37) | 8.96 (40.82) |
| Outpatient procedures | 459 | 0.03 (0.20) | 4.00 (27.78) | 479 | 0.05 (0.31) | 6.43 (39.65) |
| Inpatient stay | 459 | 0 | 0.00 | 479 | 0.00 (0.09) | 7.10 (155.49) |
| Accident and emergency (A&E) visits | 459 | 0.00 (0.09) | 0.69 (14.84) | 479 | 0.01 (0.08) | 0.99 (12.56) |
| Total intervention delivery costs | 524 | | 31.48 (10.16) | - | | 0.00 |
| Total intervention training costs | 524 | | 7.66 | - | | 0.00 |
| Total adjusted primary care consultation costs[a] | 478 | | £174.48 (£142.80 to £206.16) | 544 | | £205.61 (£166.05 to £245.18) |
| Total adjusted medication costs[a] | 478 | | £40.49 (£28.28 to £52.71) | 544 | | £46.39 (£34.90 to £57.88) |



For peer review only - http://bmjopen.bmj.com/site/about/guidelines.xhtml

| | | | | |
|---|---|---|---|---|
| Total adjusted secondary care consultation cost[a,b] | 459 | £13.78 (£0.37 to £27.19) | 479 | £25.83(£0.06 to £51.59) |

[a]Adjusted for centre, practice-size, area-level deprivation and general practice IT system; [b]Zero day case visits were reported in either arm.

Table 3. Mean unadjusted EQ-5D-5L health index score, QALYs and EQ-VAS score by trial arm

| Time period | Intervention | | Usual care | |
|---|---|---|---|---|
| | Mean(SD) [a] | n | Mean(SD) [a] | n |
| EQ-5D-5L health index score and QALYs | | | | |
| Baseline | 0.83 (0.17) | 522 | 0.83 (0.16) | 547 |
| 6 months | 0.84 (0.17) | 483 | 0.84 (0.16) | 509 |
| 12 months | 0.83 (0.17) | 457 | 0.83 (0.17) | 482 |
| Total QALYs | 0.84 (0.16) | 444 | 0.84 (0.15) | 460 |
| EQ-VAS score | | | | |
| Baseline | 78.05 (15.27) | 522 | 77.81 (15.49) | 551 |
| 6 months | 77.87 (15.93) | 481 | 76.34 (16.30) | 505 |
| 12 months | 77.13 (16.48) | 463 | 76.11 (16.46) | 481 |

[a]Higher scores represents better health

**Cost-effectiveness analysis**

As shown in Table 4, over the 12-month analysis period intervention and usual care arms had similar mean adjusted costs and QALYs. Compared to usual care, adjusted costs were -£29.99 (95% CI -£109.84 to £22.63) lower and adjusted QALYs were only 0.001 (95% CI -0.011 to 0.014) greater in the intervention arm. The positive INMB statistic (£48.01, 95% CI's: -£225.83 to £321.85) indicates the intervention is cost-effective when applying the UK's recommended threshold of £20,000 per QALY, but the overlapping confidence intervals indicates uncertainty. Similarly, the cost-effectiveness acceptability curve suggests a 63% probability of being cost-effective at this threshold (Figure 1).

**Sensitivity analysis**

In general, the sensitivity analyses (Table 4) showed similar results to the base-case analysis, with a small positive INMB and wide confidence intervals observed. However, two sensitivity analyses exploring ways to handle missing data approaches (multiple imputation, and assuming zero costs and higher QALYs for missing self-report data) led to small negative INMB and wide confidence intervals. A sensitivity analysis assuming highest costs and lower QALYs for missing self-report data, led to a positive INMB as well as wide confidence intervals. Lastly, a significant reduction in cost (-£70 CIs: -£154 to -£18) was observed when intervention costs were excluded.

**Subgroup analysis**

More than half of participants in the intervention arm (57.1%, n=236) had completed their 12-month follow up before 11th March 2020 (Table 1, Supplementary Material) (beginning of the COVID-19 pandemic). This was the case for a third of participants (37.3%, n=169) in the usual care arm. For the pre-11th March participants costs in each arm were similar compared with the primary analysis. However, QALYs were slightly lower in the intervention arm, which resulted in a negative INMB (-



For peer review only - http://bmjopen.bmj.com/site/about/guidelines.xhtml

£156, 95% CIs: -£730 to £419). Post 11th March 2020, participants in the intervention arm had lower costs and greater QALYs, while participants in the usual care arm had slightly higher costs and fewer QALYs compared to the primary analysis, which led to an overall higher INMB of £640 (95% CIs -£1445 to £2727).

For peer review only

Table 4. Primary analysis and sensitivity analyses

| Trial arm | n | Adjusted*, mean (95% CI)** Costs (£) | QALYs | Incremental adjusted mean (95% CI **) Costs (£) | QALYs | INMB (£) at £20,000/QALY (95% CI) |
|---|---|---|---|---|---|---|
| *Primary analysis: Complete Case Analysis with MLM* | | | | | | |
| Intervention | 413 | £253.53 (£215.85 to £291.22) | 0.84 (0.83 to 0.84) | -£29.99 (-£109.84 to £22.63) | 0.001 (-0.011 to 0.014) | £48.01 (-£225.83 to £321.85) |
| Usual care | 453 | £283.52 (£235.40 to £331.64) | 0.84 (0.83 to 0.84) | | | |
| *Alternative analysis: Complete Case Analysis with SUREG* | | | | | | |
| Intervention | 413 | £258.05 (£229.90 to £286.21) | 0.84 (0.83 to 0.84) | -£20.00 (-£62.50 to £22.50) | 0.001 (-0.010 to 0.011) | £35.54 (-£182.20 to £253.28) |
| Usual care | 453 | £278.05 (£251.42 to£304.68) | 0.84 (0.83 to 0.84) | | | |
| *Multiple Imputation with SUREG* | | | | | | |
| Intervention | 524 | £275.03 (£248.63 to £301.43) | 0.83 (0.83 to 0.84) | £1.42 (-£38.61 to £41.46) | -0.002 (-0.012 to 0.009) | -£35.42 (-£248.71 to £177.87) |
| Usual care | 553 | £273.61 (£248.26 to £298.96) | 0.84 (0.83 to 0.84) | | | |
| *Complete Case Analysis with MLM: Applying unit cost for face-to-face contacts used for telephone contacts* | | | | | | |
| Intervention | 413 | £262.52 (£222.73 to £302.32) | 0.84 (0.83 to 0.84) | -£34.81 (-£114.27 to £19.65) | 0.001 (-0.011 to 0.014) | £52.83 (-£222.33 to £327.99) |
| Usual care | 453 | £297.33 (£248.37 to £346.29) | 0.84 (0.83 to 0.84) | | | |
| *Complete Case Analysis with MLM: Medication quantities for SystmOne based on most frequently reported rather than average* | | | | | | |
| Intervention | 413 | £259.39 (£219.57 to £299.21) | 0.84 (0.83 to 0.84) | -£29.89 (-£118.22 to £24.25) | 0.001 (-0.011 to 0.014) | £47.92 (-£226.82 to £322.66) |
| Usual care | 453 | £289.28 (£239.48 to £339.08) | 0.84 (0.83 to 0.84) | | | |



For peer review only - http://bmjopen.bmj.com/site/about/guidelines.xhtml

For peer review only

| | | | | | | |
|---|---|---|---|---|---|---|
| *Complete Case Analysis with MLM: Removal of training costs* | | | | | | |
| Intervention | 413 | £245.87 (£208.19 to £283.56) | 0.84 (0.83 to 0.84) | -£37.65 (-£117.50 to £14.97) | 0.001 (-0.011 to 0.014) | £55.67 (-£218.17 to £329.51) |
| Usual care | 453 | £283.52 (£235.40 to £331.64) | 0.84 (0.83 to 0.84) | | | |
| *Complete Case Analysis with MLM: Removal of all intervention costs* | | | | | | |
| Intervention | 413 | £213.55 (£176.34 to £250.77) | 0.84 (0.83 to 0.84) | -£70.28 (-£153.84 to -£18.45) | 0.001 (-0.011 to 0.014) | £88.31 (-£185.50 to £362.12) |
| Usual care | 453 | £283.84 (£235.59 to £332.09) | 0.84 (0.83 to 0.84) | | | |
| *MLM: Simple imputation of £0 for secondary care per arm and a QALY value 10% higher than the mean QALY value per arm* | | | | | | |
| Intervention | 456 | £254.12 (£216.02 to £ 292.21) | 0.84 (0.83 to 0.85) | -£20.19 (-£91.44 to £30.56) | -0.006 (-0.020 to 0.008) | -£96.06 (-£394.16 to £202.04) |
| Usual care | 516 | £274.31 (£231.20 to £317.42) | 0.85 (0.84 to 0.86) | | | |
| *MLM: Simple imputation of the highest participant cost for secondary care per arm and a QALY value 10% lower than the mean QALY value per arm* | | | | | | |
| Intervention | 456 | £330.66 (£273.59 to £387.73) | 0.82 (0.82 to 0.83) | -£322.19 (-£435.12 to -£232.31) | -0.004 (-0.015 to 0.010) | £243.74 (-£39.00 to £526.48) |
| Usual care | 516 | £652.85 (£582.44 to £723.25) | 0.83 (0.82 to 0.84) | | | |

*Adjusted for centre, practice-size and area-level deprivation. In addition, costs were adjusted for general practice IT system and QALYs were adjusted for baseline utility.**Bootstrapped bias corrected and accelerated confidence intervals; SUR= Seemingly Unrelated Regression; INMB= Incremental Net Monetary Benefit; MLM= Multilevel model.

For peer review only



For peer review only - http://bmjopen.bmj.com/site/about/guidelines.xhtml

## Discussion

### Statement of principal findings

We report on the cost-effectiveness of a manualised and standardised intervention to treat male LUTS in primary care. From a UK NHS perspective, costs and QALYs that had accrued 12 months post-consent were largely similar across both arms. Applying a willingness to pay threshold of £20,000 per QALY (as stated in the UK's reference case) resulted in a small positive INMB and a 63% probability of the intervention being cost-effective when compared to usual care. The sensitivity analyses indicated that our base-case results were robust, with the exception of two sensitivity analyses exploring the impact of missing data.

### Strengths and weaknesses

Using EHRs to capture primary care resource use (23) resulted in very high rates (94.9%, n=1,022) of complete data. This meant we were able to conduct a complete case base-case analysis with 78.8% (n=413) and 81.9% (n=453) of participants included from the intervention and usual care arm, respectively. Our rates of complete data are higher than those reported in a recent review of missing data in economic evaluation. This found that missing data is very common in trial-based analyses, with studies typically reporting 63% of participants having complete cost-effectiveness data (24). The authors of the review attributed this issue of missing data to analysts' reliance on self-report questionnaires for measuring resource use and health economic outcomes.

A further advantage of using EHRs was the reduced burden for the participant, especially for those participants in the usual care group who gained little from trial participation. Self-report questionnaires which ask participants to recall details on when they accessed care, what care was provided and by whom, can be cognitively burdensome for participants (25). Evidence from an earlier large male urology trial which reported low levels of resource use from the patient's perspective (26), combined with the concern over participant burden, meant that the current study only took an NHS perspective.

Our EHR data derived from England's two main general practice IT systems (EMIS or SystmOne). A noteworthy limitation of using different EHR systems was that they provided different levels of detail. Specifically, the reason for a consultation and the quantities prescribed for a medication could not be extracted from practices which used the SystmOne IT system. In order to reduce any potential bias from using a different IT system, a standardised approach was developed. This involved including all consultations in the analysis and assigning average medication quantities reported for the EMIS cohort to the SystmOne cohort. In addition, the regression analysis adjusted for IT system and a sensitivity analysis was conducted which didn't alter the findings. Future cluster randomised studies using EHR data to capture healthcare resource use should also consider stratifying for the general practice IT system within the randomisation process, as well as controlling for this variable.

The COVID-19 pandemic also led to a number of limitations. It was not possible to request secondary care letters for all participants with missing data, because practices were reporting time constraints resulting from the pandemic. Prioritising participants with a urology referral in their EHR data was



For peer review only - http://bmjopen.bmj.com/site/about/guidelines.xhtml

therefore deemed a pragmatic approach. It was assumed the reporting quality was the same for the self-report questionnaires and secondary care letters. One practice site in the intervention arm did not provide EHRs for any of their patients (n=29) taking part in the study because of time and staff constraints resulting from the COVID-19 pandemic.

It was expected that key important and relevant differences between arms for costs and effects would be captured over the 12-month time horizon. Nevertheless, as shown in our post hoc subgroup analysis (Table 1, Supplementary Material) there were a significant proportion of participants, specifically in the usual care arm, who had their 12-month data collected during the COVID-19 pandemic. It is unclear how the pandemic impacted our analyses. In the wider literature, it has been estimated that there was around a 30% reduction in GP consultations from March 2020 up to June 2020 (27). Therefore, participants whose 12-month follow up occurred after 11th March 2020 may have reduced healthcare use due to the impact the pandemic had on primary and secondary care services in the UK. Since a larger proportion of usual care participants had their 12-month data collected during the pandemic, it is possible that resource use was underestimated in the usual care arm. Lastly, the subgroup analysis indicated higher costs for the usual care participants whose 12-month follow-up occurred during the pandemic, though the wide confidence intervals suggest this is likely to be due to chance.

Missing data in our study was primarily driven by incomplete self-report questionnaire data for secondary care activity and EQ-5D-5L data, and when this missing data was accounted for using multiple imputation analysis it resulted in the intervention arm having slightly greater costs and lower QALYs than the usual care arm, indicating the similarity of costs and effects between the two arms.

**Implications for practice**

Our study presents the first economic evaluation carried out alongside a large multi-centre definitive trial to provide evidence on the value for money of providing a manualised and standardised non-pharmacological intervention in general practice. An earlier smaller trial evaluating self-management for bothersome uncomplicated LUTS did not report on the cost-effectiveness of this type of intervention (4).

Conservative management for LUTS, as recommended by NICE, is not standardised across UK general practice. Furthermore, an audit by the Royal College of Physicians (RCP) found most men are not receiving any conservative management for their bothersome LUTS at initial assessments (28). The consequence of not implementing NICE guidelines could mean men are more likely to endure a reduction in quality of life, continue to call on primary care support due to persistent symptoms, be prescribed prostate medication or be referred to secondary care.

In the UK, an average GP consultation is around 12 minutes (29), meaning there is limited opportunity for GPs to deliver conservative management, as recommended in NICE guidelines. Our study indicates that it is possible for other primary care health care professionals to implement a standardised conservative intervention which provides advice and behavioural techniques to men at a low cost. Not including the intervention costs (£39) in the analysis led to a significant difference in



For peer review only - http://bmjopen.bmj.com/site/about/guidelines.xhtml

costs of £70 in favour of the intervention arm indicating that if costs of implementing the intervention could be reduced further for example by reducing the number of follow up contacts, as proposed in the effectiveness evaluation, then the rollout of this intervention could potentially lead to greater cost savings to the NHS.

**Conclusion**

The cost-effectiveness analysis of the TRIUMPH study showed that there were similar costs and outcomes between the two arms of the trial, indicating that the provision of a manualised and standardised non-pharmacological intervention in general practice can be implemented at low cost. This evidence in conjunction with the improvement shown in the effectiveness study, gives support to its implementation within primary care services in the UK.

For peer review only

### References


1. National Institute for Health and Care Excellence. Lower urinary tract symptoms in men: management. Clinical Guideline 97 2010 [cited 2021 28th October]. Available from: https://www.nice.org.uk/guidance/cg97
2. Hunter DJ, McKee M, Black NA, Sanderson CF. Health status and quality of life of British men with lower urinary tract symptoms: results from the SF-36. Urology. 1995;45(6):962-71.
3. National Institute for H, Care E. Lower Urinary Tract Symptoms in Men: Management [CG97]. London: NICE; 2010.
4. Brown CT, Yap T, Cromwell DA, Rixon L, Steed L, Mulligan K, et al. Self management for men with lower urinary tract symptoms: randomised controlled trial. Bmj. 2007;334(7583):25.
5. Frost J, Lane JA, Cotterill N, Fader M, Hackshaw-McGeagh L, Hashim H, et al. TReatIng Urinary symptoms in Men in Primary Healthcare using non-pharmacological and non-surgical interventions (TRIUMPH) compared with usual care: study protocol for a cluster randomised controlled trial. Trials. 2019;20(1):546.
6. NICE. Developing NICE guidelines: the manual. 7 Incorporating economic evaluation2014 [cited 2014 31st October ]. Available from: https://www.nice.org.uk/process/pmg20/chapter/incorporating-economic-evaluation.
7. van Hout B, Janssen MF, Feng Y-S, Kohlmann T, Busschbach J, Golicki D, et al. Interim Scoring for the EQ-5D-5L: Mapping the EQ-5D-5L to EQ-5D-3L Value Sets. Value in Health. 2012;15(5):708-15.
8. Drummond MF, Sculpher MJ, Claxton K, Stoddart GL, Torrance GW. Methods for the Economic Evaluation of Health Care Programmes. Oxford: Oxford: Oxford University Press; 2015.
9. Curtis L, Burns A. Unit Costs of Health and Social Care 2019 Canterbury: Personal Social Services Research Unit; 2020 [cited 2020 17th August]. Available from: https://www.pssru.ac.uk/project-pages/unit-costs/unit-costs-2019/.
10. Pope C, Turnbull J, Jones J, Prichard J, Rowsell A, Halford S. Has the NHS 111 urgent care telephone service been a success? Case study and secondary data analysis in England. BMJ Open. 2017;7(5):e014815.
11. University of Bristol Human Resources. Professorial salary ranges history 2018/19 [cited 2021 2nd March]. Available from: http://www.bristol.ac.uk/hr/salaries/gradem-prof-history.html#a1819.
12. University of Bristol Print Service. Bespoke Printing 2018 [cited 2021 2nd March ]. Available from: http://www.bristol.ac.uk/print-services/bespoke-print-promotional-products-and-signage/.
13. BT. BT Public Sector: Higher education IT solutions 2018/19 [cited 2021 2nd March]. Available from: https://business.bt.com/public-sector/universities/.
14. HM Revenue and Customs. Travel – Mileage and Fuel Rates and Allowances 2019 [cited 2021 2nd March 2021]. Available from: https://www.gov.uk/government/publications/rates-and-allowances-travel-mileage-and-fuel-allowances/travel-mileageand-fuel-rates-and-allowances.
15. National Cost Collection for the NHS. 2018/19 National Cost Collection data: NHS Improvement,; 2020 [cited 2020 17th August]. Available from: https://improvement.nhs.uk/resources/national-cost-collection/.
16. NHS Buisness Services Authority. Prescription Cost Analysis- England 2019: NHS Buisness Services Authority; 2020 [cited 2020 17th August]. Available from: https://www.nhsbsa.nhs.uk/statistical-collections/prescription-cost-analysis/prescription-cost-analysis-england-2019.
17. NHS Business Services Authority. NHS Drug Tariff 2019 [cited 2021 2nd March]. Available from: https://www.nhsbsa.nhs.uk/pharmacies-gp-practices-and-appliance-contractors/drug-tariff.
18. Curtis L, Burns A. Unit Costs of Health and Social Care 2015 Canterbury: University of Kent; 2015 [cited 2020 17th August]. Available from: https://www.pssru.ac.uk/project-pages/unit-costs/unit-costs-2015/.


For peer review only

19. Cochrane M, Noble SM, Worthington JM, Drake MJ. The TRIUMPH Study: Health Economic Analysis Plan. 2021.
20. Manca A, Hawkins N, Sculpher MJ. Estimating mean QALYs in trial-based cost-effectiveness analysis: the importance of controlling for baseline utility. Health economics. 2005;14(5):487-96.
21. El Alili M, van Dongen JM, Goldfeld KS, Heymans MW, van Tulder MW, Bosmans JE. Taking the Analysis of Trial-Based Economic Evaluations to the Next Level: The Importance of Accounting for Clustering. PharmacoEconomics. 2020;38(11):1247-61.
22. White IR, Royston P, Wood AM. Multiple imputation using chained equations: Issues and guidance for practice. Statistics in medicine. 2011;30(4):377-99.
23. Kwakkenbos L, Imran M, McCall SJ, McCord KA, Fröbert O, Hemkens LG, et al. CONSORT extension for the reporting of randomised controlled trials conducted using cohorts and routinely collected data (CONSORT-ROUTINE): checklist with explanation and elaboration. BMJ. 2021;373:n857.
24. Leurent B, Gomes M, Carpenter JR. Missing data in trial-based cost-effectiveness analysis: An incomplete journey. Health economics. 2018;27(6):1024-40.
25. Thorn JC, Brookes ST, Ridyard C, Riley R, Hughes DA, Wordsworth S, et al. Core Items for a Standardized Resource Use Measure: Expert Delphi Consensus Survey. Value Health. 2018;21(6):640-9.
26. Lewis AL, Young GJ, Selman LE, Rice C, Clement C, Ochieng CA, et al. Urodynamics tests for the diagnosis and management of bladder outlet obstruction in men: the UPSTREAM non-inferiority RCT. 2020;24:42.
27. Watt T, Firth Z, Fisher R, Thorlby R, Kelly E. Use of primary care during the COVID-19 pandemic: Patient-level data analysis of the impact of COVID-19 on primary care activity in England 2020 [cited 2021 30th November 2021]. Available from: https://www.health.org.uk/news-and-comment/charts-and-infographics/use-of-primary-care-during-the-covid-19-pandemic.
28. Gibson W, Harari D, Husk J, Lowe D, Wagg A. A national benchmark for the initial assessment of men with LUTS: data from the 2010 Royal College of Physicians National Audit of Continence Care. World J Urol. 2016;34(7):969-77.
29. Jepson M, Salisbury C, Ridd MJ, Metcalfe C, Garside L, Barnes RK. The 'One in a Million' study: creating a database of UK primary care consultations. British Journal of General Practice. 2017;67(658):e345.

For peer review only

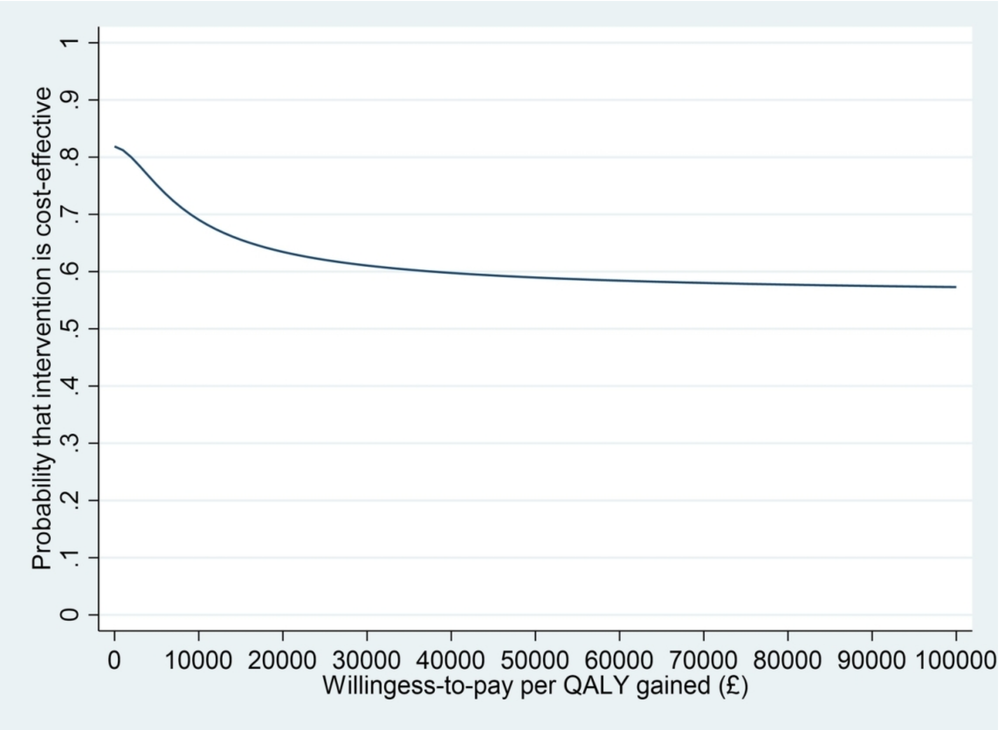


121x88mm (300 x 300 DPI)

Table 1. Additional sensitivity and subgroup analyses

| Trial arm | n | Adjusted*, mean (95% CI)** Costs (£) | QALYs | Incremental adjusted mean (95% CI **) Costs (£) | QALYs | INMB (£) at £20,000/QALY (95% CI) |
|---|---|---|---|---|---|---|
| *Complete Case Analysis with MLM: Adjusting for 12-month pre-consent GP consultation visits* | | | | | | |
| Intervention | 413 | £277.53 (£245.93 to £309.14) | 0.84 (0.83 to 0.84) | -£3.02 (-£49.52 to £44.02) | 0.001 (-0.011 to 0.014) | £21.50 (-£246.93 to £289.03) |
| Usual care | 453 | £280.56 (£244.16 to £316.96) | 0.84 (0.83 to 0.84) | | | |
| *Complete Case Analysis with MLM: Subgroup analysis for participants completing 12-month follow up before 11th March 2020* | | | | | | |
| Intervention | 236 | £255.12 (£133.88 to £376.35) | 0.83 (0.81 to 0.84) | -£33.04 (-£209.94 to £438.39) | -0.001 (-0.027 to 0.010) | £48.01 (-£225.83 to £321.85) |
| Usual care | 169 | £288.16 (£23.20 to £553.11) | 0.84 (0.82 to 0.85) | | | |
| *Complete Case Analysis with MLM: Subgroup analysis for participants completing 12-month follow up from 11th March 2020* | | | | | | |
| Intervention | 177 | £229.31 (£119.77 to £338.85) | 0.86 (0.81 to 0.90) | -£67.77 (-£219.86 to £74.92) | 0.029 (0.002 to 0.133) | £35.54 (-£182.20 to £253.28) |
| Usual care | 284 | £297.07 (£204.54 to £389.61) | 0.83 (0.76 to 0.90) | | | |

*Adjusted for centre, practice-size and area-level deprivation. In addition, costs were adjusted for general practice IT system and QALYs were adjusted for baseline utility.**Bootstrapped bias corrected and accelerated confidence intervals; SUR= Seemingly Unrelated Regression; INMB= Incremental Net Monetary Benefit; MLM= Multilevel model.

For peer review only

# CHEERS 2022 Checklist

| Topic | No. | Item | Location where item is reported |
|---|---|---|---|
| **Title** | | | |
| | 1 | Identify the study as an economic evaluation and specify the interventions being compared. | Title |
| **Abstract** | | | |
| | 2 | Provide a structured summary that highlights context, key methods, results, and alternative analyses. | Abstract |
| **Introduction** | | | |
| **Background and objectives** | 3 | Give the context for the study, the study question, and its practical relevance for decision making in policy or practice. | Introduction |
| **Methods** | | | |
| **Health economic analysis plan** | 4 | Indicate whether a health economic analysis plan was developed and where available. | Methods: Analysis section, First paragraph |
| **Study population** | 5 | Describe characteristics of the study population (such as age range, demographics, socioeconomic, or clinical characteristics). | Methods: Target population and setting section, First paragraph |
| **Setting and location** | 6 | Provide relevant contextual information that may influence findings. | Methods: Target population and setting section, First paragraph |
| **Comparators** | 7 | Describe the interventions or strategies being compared and why chosen. | Methods: Comparators section, First paragraph |
| **Perspective** | 8 | State the perspective(s) adopted by the study and why chosen. | Introduction, second paragraph |
| **Time horizon** | 9 | State the time horizon for the study and why appropriate. | Introduction, second paragraph |
| **Discount rate** | 10 | Report the discount rate(s) and reason chosen. | Methods: Analysis section, First paragraph |

| Topic | No. | Item | Location where item is reported |
|---|---|---|---|
| **Selection of outcomes** | 11 | Describe what outcomes were used as the measure(s) of benefit(s) and harm(s). | Methods: Outcomes section, First paragraph |
| **Measurement of outcomes** | 12 | Describe how outcomes used to capture benefit(s) and harm(s) were measured. | Methods: Outcomes section, First paragraph |
| **Valuation of outcomes** | 13 | Describe the population and methods used to measure and value outcomes. | Methods: Outcomes section, First paragraph |
| **Measurement and valuation of resources and costs** | 14 | Describe how costs were valued. | Methods: Resource use data collection and valuation section |
| **Currency, price date, and conversion** | 15 | Report the dates of the estimated resource quantities and unit costs, plus the currency and year of conversion. | Methods, Table 1 |
| **Rationale and description of model** | 16 | If modelling is used, describe in detail and why used. Report if the model is publicly available and where it can be accessed. | Not applicable |
| **Analytics and assumptions** | 17 | Describe any methods for analysing or statistically transforming data, any extrapolation methods, and approaches for validating any model used. | Methods, Analysis section, first and second paragraph |
| **Characterising heterogeneity** | 18 | Describe any methods used for estimating how the results of the study vary for subgroups. | Methods, Analysis section, last paragraph |
| **Characterising distributional effects** | 19 | Describe how impacts are distributed across different individuals or adjustments made to reflect priority populations. | Not applicable |
| **Characterising uncertainty** | 20 | Describe methods to characterise any sources of uncertainty in the analysis. | Methods: Analysis section |
| **Approach to engagement with patients and others affected by the study** | 21 | Describe any approaches to engage patients or service recipients, the general public, communities, or stakeholders (such as clinicians or payers) in the design of the study. | Methods: Patient and public involvement section, first paragraph |
| **Results** | | | |
| **Study parameters** | 22 | Report all analytic inputs (such as values, ranges, references) including uncertainty or distributional assumptions. | Not applicable as no model |

| Topic | No. | Item | Location where item is reported |
|---|---|---|---|
| **Summary of main results** | 23 | Report the mean values for the main categories of costs and outcomes of interest and summarise them in the most appropriate overall measure. | Results: Tables 2, 3 and 4 |
| **Effect of uncertainty** | 24 | Describe how uncertainty about analytic judgments, inputs, or projections affect findings. Report the effect of choice of discount rate and time horizon, if applicable. | Results: sensitivity analysis section |
| **Effect of engagement with patients and others affected by the study** | 25 | Report on any difference patient/service recipient, general public, community, or stakeholder involvement made to the approach or findings of the study | Methods: Patient and public involvement section, first paragraph |
| **Discussion** | | | |
| **Study findings, limitations, generalisability, and current knowledge** | 26 | Report key findings, limitations, ethical or equity considerations not captured, and how these could affect patients, policy, or practice. | Discussion |
| **Other relevant information** | | | |
| **Source of funding** | 27 | Describe how the study was funded and any role of the funder in the identification, design, conduct, and reporting of the analysis | Role of funding source, page 3 |
| **Conflicts of interest** | 28 | Report authors conflicts of interest according to journal or International Committee of Medical Journal Editors requirements. | Competing interests declaration, page 3 |

*From:* Husereau D, Drummond M, Augustovski F, et al. Consolidated Health Economic Evaluation Reporting Standards 2022 (CHEERS 2022) Explanation and Elaboration: A Report of the ISPOR CHEERS II Good Practices Task Force. Value Health 2022;25. doi:10.1016/j.jval.2021.10.008



For peer review only - http://bmjopen.bmj.com/site/about/guidelines.xhtml

BMJ Open

# Cost-effectiveness of a primary healthcare intervention to treat male lower urinary tract symptoms: the TRIUMPH cluster randomised controlled trial



| | |
|---|---|
| Journal: | *BMJ Open* |
| Manuscript ID | bmjopen-2023-075704.R1 |
| Article Type: | Original research |
| Date Submitted by the Author: | 24-Nov-2023 |
| Complete List of Authors: | Cochrane, Madeleine; University of Bristol, Population Health Sciences<br>Drake, Marcus J.; Imperial College London, Department of Surgery & Cancer<br>Worthington, Jo; University of Bristol, Population Health Sciences<br>Frost, Jessica; University of Bristol, Population Health Sciences<br>Cotterill, Nikki; University of the West of England, Department of Nursing & Midwifery<br>Fader, Mandy; University of Southampton, School of Health Sciences<br>McGeagh, Lucy ; Oxford Brookes University, Faculty of Health and Life Sciences<br>Hashim, Hashim; North Bristol NHS Trust Southmead Hospital, Bristol Urological Institute<br>Lane, Athene; University of Bristol, Population Health Sciences<br>Macaulay, Margaret; University of Southampton, Faculty of Health Sciences<br>MacNeill , Stephanie; University of Bristol, Population Health Sciences<br>Rees, Jonathan; Brockway Medical Centre<br>Ridd, Matthew; University of Bristol, Population Health Sciences<br>Robles, Luke; University of Bristol, NIHR Bristol Biomedical Research Centre<br>Sanderson, Emily; University of Bristol, Population Health Sciences<br>Taylor, Gordon; Public and Patient Involvement Representative<br>Taylor, Jodi; University of Bristol, Population Health Sciences<br>Noble, Sian; University of Bristol, Population Health Sciences |
| <b>Primary Subject Heading</b>: | Health economics |
| Secondary Subject Heading: | Urology |
| Keywords: | HEALTH ECONOMICS, UROLOGY, Primary Health Care |
| | |



SCHOLARONE™
Manuscripts

I, the Submitting Author has the right to grant and does grant on behalf of all authors of the Work (as defined in the below author licence), an exclusive licence and/or a non-exclusive licence for contributions from authors who are: i) UK Crown employees; ii) where BMJ has agreed a CC-BY licence shall apply, and/or iii) in accordance with the terms applicable for US Federal Government officers or employees acting as part of their official duties; on a worldwide, perpetual, irrevocable, royalty-free basis to BMJ Publishing Group Ltd ("BMJ") its licensees and where the relevant Journal is co-owned by BMJ to the co-owners of the Journal, to publish the Work in this journal and any other BMJ products and to exploit all rights, as set out in our licence.

The Submitting Author accepts and understands that any supply made under these terms is made by BMJ to the Submitting Author unless you are acting as an employee on behalf of your employer or a postgraduate student of an affiliated institution which is paying any applicable article publishing charge ("APC") for Open Access articles. Where the Submitting Author wishes to make the Work available on an Open Access basis (and intends to pay the relevant APC), the terms of reuse of such Open Access shall be governed by a Creative Commons licence – details of these licences and which Creative Commons licence will apply to this Work are set out in our licence referred to above.

Other than as permitted in any relevant BMJ Author's Self Archiving Policies, I confirm this Work has not been accepted for publication elsewhere, is not being considered for publication elsewhere and does not duplicate material already published. I confirm all authors consent to publication of this Work and authorise the granting of this licence.

For peer review only

**Cost-effectiveness of a primary healthcare intervention to treat male lower urinary tract symptoms: the TRIUMPH cluster randomised controlled trial**

Madeleine Cochrane* (0000-0003-1856-3293), Marcus J. Drake (0000-0002-6230-2552), Jo Worthington (0000-0002-2860-3511), Jessica Frost (0000-0002-2454-311X), Nikki Cotterill (0000-0001-6921-2712), Mandy Fader (0000-0002-0801-543X), Lucy McGeagh (0000-0002-9226-5115), Hashim Hashim (0000-0003-2467-407X), J Athene Lane (0000-0002-7578-4925), Margaret Macaulay (0000-0003-1737-4589), Stephanie J MacNeill (0000-0001-6553-1433), Jonathan Rees (0000-0001-8014-4407), Matthew J Ridd (0000-0002-7954-8823), Luke A Robles (0000-0003-2882-9868), Emily Sanderson (0000-0003-2268-4194), Gordon Taylor (0000-0002-8029-2503), Jodi Taylor (0000-0001-7171-8923), Sian Noble (0000-0002-8011-0722)

Population Health Sciences, Bristol Medical School, University of Bristol, 1-5 Whiteladies Road, Bristol, BS8 1NU, UK. Madeleine Cochrane, Senior Research Associate in Health Economics, J Athene Lane, Professor of Trials Research, Jo Worthington, Senior Trial Manager, Jessica Frost, Trial Manager, Stephanie J MacNeill, Senior Lecturer in Medical Statistics, Emily Sanderson, Research Associate in Medical Statistics, Jodi Taylor, Head of Trial Management and Operations (Bristol Trials Centre), Sian Noble, Senior Lecturer in Health Economics

Faculty of Medicine, Department of Surgery & Cancer, Imperial College London, London W12 0HS, UK. Marcus J. Drake, Chair in Neurological Urology

Department of Nursing & Midwifery, Faculty of Health & Applied Sciences, University of the West of England, Bristol, UK. Nikki Cotterill, Associate Professor in Continence Care

School of Health Sciences, University of Southampton, University Road, Southampton, SO17 1BJ, UK. Mandy Fader, Professor of Continence Technology, Margaret Macaulay, Senior Research Nurse

Bristol Urological Institute, Level 3, Learning and Research Building, North Bristol NHS Trust, Southmead Hospital, Bristol, BS10 5NB, UK. Hashim Hashim, Professor, Consultant Urological Surgeon

NIHR Bristol Biomedical Research Centre, University Hospitals Bristol and Weston NHS Foundation Trust and the University of Bristol, Level 3, University Hospitals Bristol Education Centre, Upper Maudlin Street, Bristol, BS2 8AE, UK. Luke A Robles, Senior Research Associate in Cancer

Oxford Institute Nursing, Midwifery and Allied Health Research, Oxford Brookes University, OX3 0BP. Lucy McGeagh, Senior Research Fellow

Tyntesfield Medical Group, Brockway Medical Centre, 8 Brockway, Nailsea, Bristol, BS48 1BZ, UK Jonathan Rees, General Practitioner Partner

Public and patient involvement representative, UK. Gordon Taylor, PPI representative



For peer review only - http://bmjopen.bmj.com/site/about/guidelines.xhtml

Centre of Academic Primary Care, Population Health Sciences, Bristol Medical School, University of Bristol, Canynge Hall, 39 Whatley Road, Bristol, BS8 2PS, UK. Matthew J Ridd, Professor of Primary Health Care

*Correspondence to: Dr Madeleine Cochrane: ORCiD ID 0000-0003-1856-3293; madeleine.cochrane@bristol.ac.uk; Bristol Trials Centre, Population Health Sciences, Bristol Medical School, University of Bristol, Canynge Hall, 39 Whatley Road, Bristol, BS8 2PS, UK

## Transparency statement

Cochrane had full access to all the data in the study and affirms that the manuscript is an honest, accurate, and transparent account of the study being reported; that no important aspects of the study have been omitted; and that any discrepancies from the study as originally planned (and, if relevant, registered) have been explained.

## Copyright/license for publication

The Corresponding Author has the right to grant on behalf of all authors and does grant on behalf of all authors, a worldwide licence to the Publishers and its licensees in perpetuity, in all forms, formats and media (whether known now or created in the future), to i) publish, reproduce, distribute, display and store the Contribution, ii) translate the Contribution into other languages, create adaptations, reprints, include within collections and create summaries, extracts and/or, abstracts of the Contribution, iii) create any other derivative work(s) based on the Contribution, iv) to exploit all subsidiary rights in the Contribution, v) the inclusion of electronic links from the Contribution to third party material where-ever it may be located; and, vi) licence any third party to do any or all of the above.

## Disclaimer

The views expressed in this article are those of the authors and do not necessarily reflect the opinions of the National Health Service, the National Institute for Health Research, or the Department of Health.

## Ethical approval

Approval from the NRES North West Preston Ethics Committee (18/NW/0135) was received on 11 April 2018 and applied to all NHS sites who took part in the study. All participants provided their written, informed consent to participation before entering the study

## Dissemination

TRIUMPH study participants will be informed of the study results via our study website (bristol.ac.uk/triumph-study). In addition, they will be sent details of the results via the study newsletter.



For peer review only - http://bmjopen.bmj.com/site/about/guidelines.xhtml

## Acknowledgements

This study was designed and delivered in collaboration with the Bristol Randomised Trials Collaboration (BRTC), a UKCRC registered clinical trials unit which, as part of the Bristol Trials Centre, is in receipt of National Institute for Health Research CTU support funding. The University of Bristol acted as the Sponsor for this trial and the trial was hosted by the NHS Bristol, North Somerset and South Gloucestershire Clinical Commissioning Group (CCG). The TRIUMPH Research team acknowledges the support of the National Institute for Health Research Clinical Research Network (NIHR CRN). Study data were collected and managed using REDCap hosted at the University of Bristol.

The authors would like to thank all participants, principal investigators and their teams at each of the TRIUMPH study sites for their involvement, and the West of England and Wessex CRNs for their role in the study. The authors would also like to thank the members of the Patient Advisory Group, Trial Steering Committee and Data Monitoring Committee.

## Trial registration

Trial registration was prospective before enrolling the first patient. ISRCTN registry number: ISRCTN11669964. Registration date: 12/04/2018.



For peer review only - http://bmjopen.bmj.com/site/about/guidelines.xhtml

## Abstract

**Objectives:** To estimate the cost-effectiveness of a primary care intervention for male lower urinary tract symptoms (LUTS) compared with usual care.

**Design:** Economic evaluation alongside a cluster randomised controlled trial from an UK National Health Service (NHS) perspective with a 12-month time horizon.

**Setting:** Thirty NHS general practice sites in England.

**Participants:** 1,077 men aged 18 or older identified in primary care with bothersome LUTS.

**Interventions:** A standardised and manualised intervention for the treatment of bothersome LUTS was compared to usual care. The intervention group (n=524) received a standardised information booklet with guidance on conservative treatment for LUTS, urinary symptom assessment, and follow up contacts for 12 weeks. The usual care group (n=553) followed local guidelines between general practice sites.

**Measures:** Resource use was obtained from electronic health records, trial staff and participants, and valued using UK reference costs. Quality-adjusted life years (QALYs) were calculated from the EQ-5D-5L questionnaire. Adjusted mean differences in costs and QALYs and incremental net monetary benefit were estimated.

**Results:** 866 of 1,077 (80.4%) participants had complete data and were included in the base-case analysis. Over the 12-month follow-up period, intervention and usual care arms had similar mean adjusted costs and QALYs. Mean differences were lower in the intervention arm for adjusted costs -£29.99 (95% CI -£109.84 to £22.63) while higher in the intervention arm for adjusted QALYs 0.001 (95% CI -0.011 to 0.014). The incremental net monetary benefit statistic was £48.01 (95% CI -£225.83 to £321.85) at the NICE UK threshold of £20,000 per QALY. The cost-effectiveness acceptability curve showed a 63% probability of the intervention arm being cost-effective at this threshold.

**Conclusions:** Costs and QALYs were similar between the two arms at 12 months follow-up. This indicates that the intervention can be implemented in general practice at neutral cost.

**Trial registration:** ISRCTN11669964.




For peer review only - http://bmjopen.bmj.com/site/about/guidelines.xhtml

## Strengths and limitations of this study

- This was a multi-centre study that collected data from a large number of general practice sites and participants.
- A strength of using electronic health records was that it reduced the data collection and recall burden for participants.
- A limitation of using electronic health records was that different levels of detail were provided by the two general practice IT systems.

For peer review only



For peer review only - http://bmjopen.bmj.com/site/about/guidelines.xhtml

## Introduction

Lower urinary tract symptoms (LUTS) is a bothersome condition among the ageing male population with a prevalence of up to 30% in men over 65 years old (1). LUTS includes storage, voiding and post-micturition symptoms, and can have a considerable impact on quality of life (2). Causes of LUTS include benign prostate enlargement (BPE), detrusor muscle weakness or overactivity, prostate inflammation and lifestyle habits. Evidence-based national guidelines from the UK's National Institute for Health and Care Excellence (NICE) recommend conservative management interventions (such as fluid and caffeine intake, bladder training, urethral compression and release, and pelvic floor muscle exercises) at initial assessments for LUTS, once serious underlying conditions (e.g. urinary tract infection, prostate cancer, neurological disease) have been excluded (1).

Conservative management options (3), are time-consuming and complex to implement due to the range of possible symptoms and causes of LUTS. Initial evidence from a small single centre randomised controlled trial (RCT) suggests conservative self-management interventions could play a role in the delivery of treatment for LUTS. However, this smaller trial only evaluated effectiveness and not cost-effectiveness (4). The present study, TReatIng Urinary symptoms in Men in Primary Healthcare (TRIUMPH), sought to provide evidence on whether the provision of a manualised and standardised non-pharmacological intervention in primary care, is effective and cost-effective compared to usual care. The effectiveness results, reported elsewhere, showed improved symptoms for men with LUTS and these benefits were sustained for a full year (5). The current cost-effectiveness study is conducted from an NHS perspective over a 12-month time horizon, with the intention to inform clinical practice decisions within primary care. The analysis is reported according to the Consolidated Health Economic Evaluation Reporting Standards (CHEERS) guidelines(6).

## Methods

### Target population and setting

Participants in the TRIUMPH study were men aged 18 or over, who considered themselves to have bothersome LUTS and had presented to primary care within the past five years with at least one symptom of LUTS. The trial was carried out across thirty general practice sites in Southwest England. The National Research Ethics Service North West Preston Ethics Committee (18/NW/0135) gave ethical approval and all study participants gave written, informed consent before participating in the study.

### Comparators

The intervention arm, a manualised and standardised intervention, involved the provision of a information booklet with guidance on conservative interventions for LUTS. Participants had an initial symptom assessment with a Nurse or Healthcare Assistant in order to understand their symptom needs, bothersomeness, and impact on their quality of life, as well as their personal circumstances. This was delivered face-to-face. Participants were then directed to specific sections of the information booklet. Participants were offered up to three further contacts from the healthcare professional. The



For peer review only - http://bmjopen.bmj.com/site/about/guidelines.xhtml

first of the three was offered via telephone while for the remaining two, participants were given the option to receive these via telephone, email or text. The comparator group continued to receive care as usual.

**Study design**

The economic evaluation was conducted alongside the TRIUMPH trial, a multicentre cluster RCT, and evaluated costs and outcomes from an NHS perspective over a 12-month time horizon. Detailed methods for the design of the TRIUMPH trial [ISRCTN11669964] are described elsewhere (7). In brief, thirty general practice sites were randomised on a 1:1 basis to either deliver the intervention or to continue care as usual. Randomisation was minimised by NIHR Clinical Research Network (CRN) centre (West of England and Wessex), practice size and area-level deprivation.

**Patient and public involvement**

A Patient Advisory Group (PAG) was set up to ensure patient and public involvement (PPI) throughout all stages of the research. PPI representatives were involved in the design of the study, ensuring all study material were usable and appropriate for a non-specialist audience. In particular, the PPI group reviewed and advised on the following study components: TRIUMPH intervention booklet, patient-facing questionnaires, newsletters, website, initial qualitative results relating to men's experiences of the patient pathways for LUTS within the NHS, and agreeing to plans for implementing and disseminating the study results. Two PPI representatives also played a significant role in planning and chairing the PAG meetings. In addition, both the Trial Management Group and Trial Steering Committee each included a patient representative who contributed to the management and conduct of the trial.

**Outcomes**

The quality-adjusted life year (QALY) was used as the outcome measure, as recommended in the UK's reference case (8) for interventions funded by the NHS. Health status was measured using the EQ-5D-5L questionnaire and EQ-5D-VAS at baseline, six and 12 months. At baseline the questionnaire was administered via post, while at six and 12 months it could be completed by post or online. Participants' EQ-5D-5L scores were mapped to the EQ-5D-3L valuation set using a validated mapping function by van Hout et al. (9). This enabled a utility score to be calculated for each participant based on published pre-existing utility scores derived from a representative sample of the UK population. Utility scores were then combined with length of life to calculate the QALYs for each participant using an area-under-the-curve approach (10). The approach accounted for any deaths that occurred during the 12-month trial period.

**Resource use data collection and valuation**

Resource use data was obtained for intervention training and delivery costs, primary care consultations and LUTS- related medication and secondary care. Data for each individual patient's 12-month follow up period was captured via the following sources: trial management records, case report forms, primary care Electronic Health Records (EHR) data and self-report questionnaires.



For peer review only - http://bmjopen.bmj.com/site/about/guidelines.xhtml

Intervention-related resources in terms of training, including staff time, travel and teleconference expenses, were obtained primarily through trial management records. Expert opinion from the Lead Nurses was used to capture duration of the training. Resources used in the delivery of the intervention in terms of staff time for the initial clinic visit and subsequent follow-up were prospectively recorded on case report forms. Primary care EHRs were used to capture: (1) all primary care consultations with a GP, Nurse, Healthcare Assistant or Pharmacist; and (2) LUTS-related prescribed medication. .Database searches were built to extract EHRs from primary care administration systems (EMIS and SystmOne). Records were extracted from each general practice site at least one month after their last recruited participant had reached their 12-month follow up date.

Secondary care was predominantly collected via patient self-report questionnaires at six and 12-month follow-up, administered either online or via post. Participants were asked to report LUTS-related outpatient, day case, inpatient and A&E visits. EHR data were used to identify secondary care referrals. If a participant had a urology referral but had missing or no secondary care self-report data, then we reviewed the participant's secondary care letters.

A urology clinician was consulted to identify LUTS-related prescribed medications and secondary care visits. More specifically, Prescribed medications were extracted from the EHRs if they were coded under the drug criteria: urinary frequency, nocturnal enuresis, incontinence, urinary retention or diuretics. The clinician reviewed the names and doses of the extracted medications and created a list of LUTS-related medications which was applied by the analyst. Similarly, the clinician reviewed a list of the types of secondary care visits reported by the participants and in the primary care letters. The clinician created a list of LUTS-related care and this was applied by the analyst.

Details of how the resources were valued are reported in Table 1. 2018/19 costs were used to value the resource use. The NHS cost inflation index (NHSCII) was used to inflate a unit cost to current prices if it was unavailable for the year of analysis (11).

**Table 1. Resources collected and their valuation**

| Resources | Unit cost (£) | Source of unit cost |
|---|---|---|
| GP Surgery visit | 34[a,b] | Curtis and Burns, 2019(11) |
| GP Telephone call | 26.27 [a,b,c] | Curtis and Burns, 2019(11) |
| GP Home visit | 87.28 [a,b,d] | Curtis and Burns, 2019(11) |
| Practice Nurse Surgery visit | 10.85 [b,e] | Curtis and Burns, 2019(11) |
| Practice Nurse Telephone call | 8.25 [b,f] | Curtis and Burns, 2019(11) |
| Healthcare Assistant Surgery visit | 6.67[e] | Curtis and Burns, 2019(11) |
| Healthcare Assistant Telephone call | 5.07[f] | Curtis and Burns, 2019(11) |
| Pharmacist Surgery visit | 11.63[e] | Curtis and Burns, 2019(11) |
| Pharmacist Surgery Telephone call | 8.84[f] | Curtis and Burns, 2019(11) |
| NHS 111 call | 12.26 | Pope et al. 2017 (12) |
| Trainers (Professor; Lead Nurses) | Varies | Research Institution's pay scales, 2018/19[g] (13); Curtis and Burns, 2019[h] (11) |
| Intervention booklet | 6.15[i] | Research Institution's printing service charge(14) |
| Intervention visit time per minute | Varies[j] | Curtis and Burns, 2019(11) |
| Follow up contact time per minute | Varies[k] | Curtis and Burns, 2019(11) |
| Teleconference call per minute | 0.15 | BT Conference Call service charge(15) |
| Car mileage | 0.45[l] | HM Revenue and Customs (16) |
| Outpatient procedures | Varies | NHS National Cost Collection, 2018/19(17) |
| Outpatient visits | 110[m] | NHS National Cost Collection, 2018/19(17) |



For peer review only - http://bmjopen.bmj.com/site/about/guidelines.xhtml

| | | |
|---|---|---|
| Day cases | Varies | NHS National Cost Collection, 2018/19(17) |
| Inpatient admissions | Varies | NHS National Cost Collection, 2018/19(17) |
| Accident & Emergency attendances | 159 | NHS National Cost Collection, 2018/19(17) |
| Medications | Varies[n] | Prescription Cost Analysis, 2019(18);<br>NHS Drug Tariff, 2019(19) |

[a]Excluding direct care staff costs
[b]Including qualifications
[c]Based on the assumption of a 7.1 minute telephone consultation, as reported in earlier unit cost series by Curtis and Burns (20)
[d]Based on the assumption of a 11.4 minute home visit and 12 minutes of travel, as reported in earlier unit cost series by Curtis and Burns (20)
[e]Based on the assumption of a 15.5 minute surgery consultation, as reported for a Practice Nurse in earlier unit cost series by Curtis and Burns. It was assumed the Nurses (practice and research), HCAs and Pharmacists had the same surgery consultation length (20)
[f]There is no published unit cost for non-triage telephone consultations for Practice Nurses, HCAs and Pharmacists. Telephone consultations were assumed to be 11.8 minutes for these healthcare professionals. This was based on data in Curtis and Burns 2015 and 2019 series, which indicates face-to-face and triage telephone consultations delivered by a Practice Nurse are approximately 1.5 times longer than those provided by a GP(11, 20).
[g]Including basic salary, National Insurance and superannuation
[h]Assuming Band 6 for all Lead Nurses
[i] All booklets had to be prepared in advance, it was therefore assumed all participants randomised to the intervention arm incurred the booklet cost
[j]For intervention visits, length of visit was reported in minutes in the case report form. Unit cost varied depending upon which staff type delivered the intervention.
[k]For telephone follow-ups, length of visit was reported in minutes in the case report form. Unit cost varied depending upon which staff type delivered the intervention. For texts and emails, the number of these were reported in the case report form. Time taken to send each text and/or email was assumed to be two minutes.
[l]Distance travelled was calculated by measuring the distance between the staff's workplace and the training locations

[m]For outpatient visits a weighted average of the unit costs for consultant and non-consultant led visits was used.

[n]In the minority of instances where a unit cost was not available in the Prescription Cost Analysis, then the unit costs from the 2019 NHS Drug Tariff were used

**Analysis**

An analysis plan was pre-specified before all data were collected and STATA version 16.1 was used for all analyses(21). The primary economic analysis was to evaluate costs and outcomes over a 12-month time horizon and so discounting was not required. EHR data were derived from two different IT systems, a standardised cleaning process was therefore implemented to ensure the final analysis dataset was comparable across the two systems. All participants were analysed in accordance with the allocation group they were assigned to at the point of randomisation.

Simple mean imputation was used for a minority of participants who had missing data in relation to their length of follow up visit at week 1 (n=1) and week 4 (n=7) in their case report forms. Additionally, it was assumed zero secondary care visits occurred if other parts of the questionnaire had been completed but the resource use questions had been left blank and the patient had no urology referrals recorded in their EHRs. Total costs were then calculated for each individual participant by summing all the costs across all categories (intervention, primary care, LUTS medications and secondary care) and mean total unadjusted costs per arm were then estimated.

A mixed effects multilevel model (MLM) using random intercepts and assuming a two-level structure was used to account for clustered data and to adjust for the minimisation variables (practice size, IMD



For peer review only - http://bmjopen.bmj.com/site/about/guidelines.xhtml

and centre) as well as general practice IT system and baseline utility for costs and QALYs (22), respectively. El Alili et al. (23) found MLMs performed better than OLS regression for cluster-based economic evaluations. The MLM was used to calculate the adjusted mean costs and QALYs, and the adjusted mean differences in costs and QALYs between groups. For both costs and QALYs, 95% CIs were estimated using bias-corrected and accelerated (BCa) bootstrapping (23).

Regression outputs from the MLM facilitated the estimation of an incremental net monetary benefit (INMB) statistic and the associated confidence intervals at NICE's recommended willingness to pay threshold of £20,000 and £30,000 per QALY (MLM outputs in Tables 1 and 2, Supplementary Material). A cost-effectiveness acceptability curve (CEAC) was constructed to present the uncertainty over a range of willingness to pay thresholds. This was done using the parametric p-value approach as applied by El Alili et al.(23) and Hoch et al.(4).

One-way sensitivity analyses were conducted to explore methodological uncertainty:

- An alternative analytic model was employed by using a seemingly unrelated regression (SUR) model instead of a MLM.
- Inconsistency in coding the mode of delivery for primary care consultations was explored by re-coding all unit costs for telephone contacts to unit costs for face-to-face contacts.
- Training costs were excluded from the total intervention costs, as it is possible that these costs are captured in the published unit costs.
- Intervention training and delivery costs were excluded.
- The primary analysis model was adjusted for GP consultation costs for the 12-month period pre-consent.
- Missing data assumptions were tested using a range of approaches, including:
    - For our primary analysis we assumed our data were missing completely at random. In addition, we tested the assumption that our data were missing at random by performing multiple imputation by chained equations with predictive mean matching and our SUR model. The covariates in the multiple imputation model were site ID, baseline utility, centre, practice size, IMD and general practice system. The model was run by allocation group and a randomisation seed was set to ensure reproducible imputations. A total of 25 imputations were performed and combined with Rubin's rule in Stata 16.1 (24).
    - For each arm, zero costs and a QALY value that was 10% higher than the mean QALY value was imputed for participants with missing questionnaire data.
    - For participants with missing questionnaire data, the largest participant cost for secondary care and a QALY value 10% lower than the mean QALY value per arm were imputed.
    - SystmOne EHR based practices did not include data on the quantity of tablets prescribed per medication. In the base-case analysis, EMIS practices data was used to estimate the mean quantity in the SystmOne practices. A sensitivity analysis used the most frequently reported quantity, rather than the mean.



For peer review only - http://bmjopen.bmj.com/site/about/guidelines.xhtml

A post-hoc subgroup analysis compared participants who had completed follow-up from 11th March 2020 (where 11th March 2020 reflects when the COVID-19 outbreak was declared internationally as a pandemic) to those who completed follow-up before this date.

**Results**

1,077 men were recruited; either to receive the intervention sites (n=524) or usual care sites (n=553). There was a low rate (5.1%, n=55) of missing EHR data (primary care and medications), and a greater rate of item missingness for the self-report secondary care (12.5%, n=135) and EQ-5D-5L (16.1%, n=173) data. This rate of missing data permitted a complete case primary analysis on 866 out of 1,077 (80.4%) participants, 413 (78.8%) and 453 (81.9%) participants in the intervention and usual care arm, respectively.

**Resource use, costs and outcomes**

Table 2 shows that mean resource use and costs from available cases were similar between arms, as were the adjusted mean costs for total primary care consultations, medications and secondary care visits. Mean intervention costs were £39, excluding intervention training costs reduced this to £31. Overall, EQ-5D-5L scores, QALYs and EQ-VAS scores were similar across both groups and all timepoints (Table 3).

Table 2. Resource use and costs from NHS perspective

| Resource category | Intervention (n=524) | | | Usual care (n=553) | | |
|---|---|---|---|---|---|---|
| | n | Resource use, mean (SD) | Cost, mean (SD) (£) | n | Resource use, mean (SD) | Cost, mean (SD) (£) |
| GP consultations all types | 478 | 4.60 (4.67) | 145.86 (162.62) | 544 | 5.00 (5.68) | 158.17 (180.05) |
| Practice Nurse consultations all types | 478 | 2.78 (3.64) | 28.77 (38.58) | 544 | 3.61 (5.75) | 38.41 (58.48) |
| HCA all types of consultations | 478 | 1.16 (2.05) | 7.70 (13.67) | 544 | 0.59 (2.07) | 3.91 (13.82) |
| Pharmacist all types of consultations | 478 | 0.15 (0.54) | 1.60 (5.59) | 544 | 0.15 (0.90) | 1.36 (7.18) |
| Total LUTS medications | 478 | 3.27 (4.74) | 45.77 (100.50) | 544 | 4.29 (5.75) | 42.22 (98.97) |
| NHS 111 encounters | 459 | 0 | 0.00 | 479 | 0.01 (0.14) | 0.08 (1.72) |
| Outpatient visits | 459 | 0.07(0.34) | 8.04(36.75) | 479 | 0.08(0.37) | 8.96 (40.82) |
| Outpatient procedures | 459 | 0.03 (0.20) | 4.00 (27.78) | 479 | 0.05 (0.31) | 6.43 (39.65) |
| Inpatient stay | 459 | 0 | 0.00 | 479 | 0.00 (0.09) | 7.10 (155.49) |
| Accident and emergency (A&E) visits | 459 | 0.00 (0.09) | 0.69 (14.84) | 479 | 0.01 (0.08) | 0.99 (12.56) |



For peer review only - http://bmjopen.bmj.com/site/about/guidelines.xhtml

| | | | | |
|---|---|---|---|---|
| Total intervention delivery costs | 524 | 31.48 (10.16) | - | 0.00 |
| Total intervention training costs | 524 | 7.66 | - | 0.00 |
| Total adjusted primary care consultation costs[a] | 478 | £174.48 (£142.80 to £206.16) | 544 | £205.61 (£166.05 to £245.18) |
| Total adjusted medication costs[a] | 478 | £40.49 (£28.28 to £52.71) | 544 | £46.39 (£34.90 to £57.88) |
| Total adjusted secondary care consultation cost[a,b] | 459 | £13.78 (£0.37 to £27.19) | 479 | £25.83(£0.06 to £51.59) |

[a]Adjusted for centre, practice-size, area-level deprivation and general practice IT system; [b]Zero day case visits were reported in either arm.

Table 3. Mean unadjusted EQ-5D-5L health index score, QALYs and EQ-VAS score by trial arm

| Time period | Intervention | | Usual care | |
|---|---|---|---|---|
| | Mean(SD)[a] | n | Mean(SD)[a] | n |
| EQ-5D-5L health index score and QALYs | | | | |
| Baseline | 0.83 (0.17) | 522 | 0.83 (0.16) | 547 |
| 6 months | 0.84 (0.17) | 483 | 0.84 (0.16) | 509 |
| 12 months | 0.83 (0.17) | 457 | 0.83 (0.17) | 482 |
| Total QALYs | 0.84 (0.16) | 444 | 0.84 (0.15) | 460 |
| EQ-VAS score | | | | |
| Baseline | 78.05 (15.27) | 522 | 77.81 (15.49) | 551 |
| 6 months | 77.87 (15.93) | 481 | 76.34 (16.30) | 505 |
| 12 months | 77.13 (16.48) | 463 | 76.11 (16.46) | 481 |

[a]Higher scores represents better health

**Cost-effectiveness analysis**

As shown in Table 4, over the 12-month analysis period intervention and usual care arms had similar mean adjusted costs and QALYs. Compared to usual care, adjusted costs were -£29.99 (95% CI -£109.84 to £22.63) lower and adjusted QALYs were only 0.001 (95% CI -0.011 to 0.014) greater in the intervention arm. The INMB results (£48.01, 95% CI's: -£225.83 to £321.85) and the cost-effectiveness acceptability curve (CEAC) suggest a 63% probability of the intervention being cost-effective when applying the UK's recommended threshold of £20,000 per QALY (Figure 1). Together the CEAC and the 95% confidence intervals of the INMB results, which include zero, indicates there is uncertainty in our findings.

**Sensitivity analysis**

In general, the sensitivity analyses (Table 4) showed similar results to the base-case analysis, with a small positive INMB and wide confidence intervals observed. However, two sensitivity analyses exploring ways to handle missing data approaches (multiple imputation, and assuming zero costs and higher QALYs for missing self-report data) led to small negative INMB and wide confidence intervals. A sensitivity analysis assuming highest costs and lower QALYs for missing self-report data, led to a

For peer review only



For peer review only - http://bmjopen.bmj.com/site/about/guidelines.xhtml

positive INMB as well as wide confidence intervals. Lastly, a £70 (95% CIs: -£154 to -£18) reduction in cost was observed when intervention costs were excluded.

**Subgroup analysis**

More than half of participants in the intervention arm (57.1%, n=236) had completed their 12-month follow up before 11th March 2020 (Table 3, Supplementary Material) (beginning of the COVID-19 pandemic). This was the case for a third of participants (37.3%, n=169) in the usual care arm. For the pre-11th March participants costs in each arm were similar compared with the primary analysis. However, QALYs were slightly lower in the intervention arm, which resulted in a negative INMB (-£156, 95% CIs: -£730 to £419). Post 11th March 2020, participants in the intervention arm had lower costs and greater QALYs, while participants in the usual care arm had slightly higher costs and fewer QALYs compared to the primary analysis, which led to an overall higher INMB of £640 (95% CIs -£1445 to £2727).

For peer review only



For peer review only - http://bmjopen.bmj.com/site/about/guidelines.xhtml

Table 4. Primary analysis and sensitivity analyses

| Trial arm | n | Adjusted*, mean (95% CI)** Costs (£) | QALYs | Incremental adjusted mean (95% CI **) Costs (£) | QALYs | INMB (£) at £20,000/QALY (95% CI) |
|---|---|---|---|---|---|---|
| *Primary analysis: Complete Case Analysis with MLM* | | | | | | |
| Intervention | 413 | £253.53 (£215.85 to £291.22) | 0.836 (0.828 to 0.845) | -£29.99 (-£109.84 to £22.63) | 0.001 (-0.011 to 0.014) | £48.01 (-£225.83 to £321.85) |
| Usual care | 453 | £283.52 (£235.40 to £331.64) | 0.836 (0.828 to 0.843) | | | |
| *Alternative analysis: Complete Case Analysis with SUREG* | | | | | | |
| Intervention | 413 | £258.05 (£229.90 to £286.21) | 0.836 (0.829 to 0.844) | -£20.00 (-£62.50 to £22.50) | 0.001 (-0.010 to 0.011) | £35.54 (-£182.20 to £253.28) |
| Usual care | 453 | £278.05 (£251.42 to£304.68) | 0.836 (0.829 to 0.842) | | | |
| *Multiple Imputation with SUREG* | | | | | | |
| Intervention | 524 | £275.03 (£248.63 to £301.43) | 0.835 (0.828 to 0.842) | £1.42 (-£38.61 to £41.46) | -0.002 (-0.012 to 0.009) | -£35.42 (-£248.71 to £177.87) |
| Usual care | 553 | £273.61 (£248.26 to £298.96) | 0.836 (0.829 to 0.843) | | | |
| *Complete Case Analysis with MLM: Applying unit cost for face-to-face contacts used for telephone contacts* | | | | | | |
| Intervention | 413 | £262.52 (£222.73 to £302.32) | 0.836 (0.828 to 0.844) | -£34.81 (-£114.27 to £19.65) | 0.001 (-0.011 to 0.014) | £52.83 (-£222.33 to £327.99) |
| Usual care | 453 | £297.33 (£248.37 to £346.29) | 0.836 (0.828 to 0.843) | | | |
| *Complete Case Analysis with MLM: Medication quantities for SystmOne based on most frequently reported rather than average* | | | | | | |
| Intervention | 413 | £259.39 (£219.57 to £299.21) | 0.836 (0.828 to 0.845) | -£29.89 (-£118.22 to £24.25) | 0.001 (-0.011 to 0.014) | £47.92 (-£226.82 to £322.66) |
| Usual care | 453 | £289.28 (£239.48 to £339.08) | 0.836 (0.828 to 0.843) | | | |

For peer review only

| | | | | | | |
|---|---|---|---|---|---|---|
| *Complete Case Analysis with MLM: Removal of training costs* | | | | | | |
| Intervention | 413 | £245.87 (£208.19 to £283.56) | 0.836 (0.828 to 0.845) | -£37.65 (-£117.50 to £14.97) | 0.001 (-0.011 to 0.014) | £55.67 (-£218.17 to £329.51) |
| Usual care | 453 | £283.52 (£235.40 to £331.64) | 0.836 (0.828 to 0.843) | | | |
| *Complete Case Analysis with MLM: Removal of all intervention costs* | | | | | | |
| Intervention | 413 | £213.55 (£176.34 to £250.77) | 0.8436 (0.828 to 0.845) | -£70.28 (-£153.84 to -£18.45) | 0.001 (-0.011 to 0.014) | £88.31 (-£185.50 to £362.12) |
| Usual care | 453 | £283.84 (£235.59 to £332.09) | 0.836 (0.828 to 0.843) | | | |
| *MLM: Simple imputation of £0 for secondary care per arm and a QALY value 10% higher than the mean QALY value per arm* | | | | | | |
| Intervention | 456 | £254.12 (£216.02 to £ 292.21) | 0.842 (0.834 to 0.850) | -£20.19 (-£91.44 to £30.56) | -0.006 (-0.020 to 0.008) | -£96.06 (-£394.16 to £202.04) |
| Usual care | 516 | £274.31 (£231.20 to £317.42) | 0.848 (0.839 to 0.857) | | | |
| *MLM: Simple imputation of the highest participant cost for secondary care per arm and a QALY value 10% lower than the mean QALY value per arm* | | | | | | |
| Intervention | 456 | £330.66 (£273.59 to £387.73) | 0.824 (0.816 to 0.833) | -£322.19 (-£435.12 to -£232.31) | -0.004 (-0.015 to 0.010) | £243.74 (-£39.00 to £526.48) |
| Usual care | 516 | £652.85 (£582.44 to £723.25) | 0.838 (0.821 to 0.836) | | | |

*Adjusted for centre, practice-size and area-level deprivation. In addition, costs were adjusted for general practice IT system and QALYs were adjusted for baseline utility.**Bootstrapped bias corrected and accelerated confidence intervals; SUR= Seemingly Unrelated Regression; INMB= Incremental Net Monetary Benefit; MLM= Multilevel model.



For peer review only - http://bmjopen.bmj.com/site/about/guidelines.xhtml

For peer review only

## Discussion

### Statement of principal findings

We report on the cost-effectiveness of a manualised and standardised intervention to treat male LUTS in primary care. From a UK NHS perspective, costs and QALYs that had accrued 12 months post-consent were largely similar across both arms. Applying a willingness to pay threshold of £20,000 per QALY (as stated in the UK's reference case) resulted in a small positive INMB and a 63% probability of the intervention being cost-effective when compared to usual care. The sensitivity analyses indicated that our base-case results were robust, with the exception of two sensitivity analyses exploring the impact of missing data.

### Strengths and weaknesses

Using EHRs to capture primary care resource use (25) resulted in very high rates (94.9%, n=1,022) of complete data. This meant we were able to conduct a complete case base-case analysis with 78.8% (n=413) and 81.9% (n=453) of participants included from the intervention and usual care arm, respectively. Our rates of complete data are higher than those reported in a recent review of missing data in economic evaluation. This found that missing data is very common in trial-based analyses, with studies typically reporting 63% of participants having complete cost-effectiveness data (26). The authors of the review attributed this issue of missing data to analysts' reliance on self-report questionnaires for measuring resource use and health economic outcomes.

A further advantage of using EHRs was the reduced burden for the participant, especially for those participants in the usual care group who gained little from trial participation. Self-report questionnaires which ask participants to recall details on when they accessed care, what care was provided and by whom, can be cognitively burdensome for participants (27). Evidence from an earlier large male urology trial which reported low levels of resource use from the patient's perspective (28), combined with the concern over participant burden, meant that the current study only took an NHS perspective.

Our EHR data derived from England's two main general practice IT systems (EMIS or SystmOne). A noteworthy limitation of using different EHR systems was that they provided different levels of detail. Specifically, the reason for a consultation and the quantities prescribed for a medication could not be extracted from practices which used the SystmOne IT system. In order to reduce any potential bias from using a different IT system, a standardised approach was developed which we have described in our methods. In the regression analysis we adjusted for IT system and a sensitivity analysis was conducted which didn't alter the findings. Future cluster randomised studies using EHR data to capture healthcare resource use should also consider stratifying for the general practice IT system within the randomisation process, as well as controlling for this variable.

The COVID-19 pandemic also led to a number of limitations. It was not possible to request secondary care letters for all participants with missing data, because practices were reporting time constraints resulting from the pandemic. Prioritising participants with a urology referral in their EHR data was therefore deemed a pragmatic approach. It was assumed the reporting quality was the same for the



For peer review only - http://bmjopen.bmj.com/site/about/guidelines.xhtml

self-report questionnaires and secondary care letters. One practice site in the intervention arm did not provide EHRs for any of their patients (n=29) taking part in the study because of time and staff constraints resulting from the COVID-19 pandemic.

It was expected that key important and relevant differences between arms for costs and effects would be captured over the 12-month time horizon. Nevertheless, as shown in our post hoc subgroup analysis (Table 3, Supplementary Material) there were a significant proportion of participants, specifically in the usual care arm, who had their 12-month data collected during the COVID-19 pandemic. It is unclear how the pandemic impacted our analyses. In the wider literature, it has been estimated that there was around a 30% reduction in GP consultations from March 2020 up to June 2020 (29). Therefore, participants whose 12-month follow up occurred after 11th March 2020 may have reduced healthcare use due to the impact the pandemic had on primary and secondary care services in the UK. Since a larger proportion of usual care participants had their 12-month data collected during the pandemic, it is possible that resource use was underestimated in the usual care arm. Lastly, the subgroup analysis indicated higher costs for the usual care participants whose 12-month follow-up occurred during the pandemic, though the wide confidence intervals suggest this is likely to be due to chance.

Missing data in our study was primarily driven by incomplete self-report questionnaire data for secondary care activity and EQ-5D-5L data, and when this missing data was accounted for using multiple imputation analysis it resulted in the intervention arm having slightly greater costs and lower QALYs than the usual care arm, indicating the similarity of costs and effects between the two arms.

**Implications for practice**

Our study presents the first economic evaluation carried out alongside a large multi-centre definitive trial to provide evidence on the value for money of providing a manualised and standardised non-pharmacological intervention in general practice. An earlier smaller trial evaluating self-management for bothersome uncomplicated LUTS did not report on the cost-effectiveness of this type of intervention (4).

Conservative management for LUTS, as recommended by NICE, is not standardised across UK general practice. Furthermore, an audit by the Royal College of Physicians (RCP) found most men are not receiving any conservative management for their bothersome LUTS at initial assessments (30). The consequence of not implementing NICE guidelines could mean men are more likely to endure a reduction in quality of life, continue to call on primary care support due to persistent symptoms, be prescribed prostate medication or be referred to secondary care.

In the UK, an average GP consultation is around 12 minutes (31), meaning there is limited opportunity for GPs to deliver conservative management, as recommended in NICE guidelines. Our study indicates that it is possible for other primary care health care professionals to implement a standardised conservative intervention which provides advice and behavioural techniques to men at a neutral cost.



For peer review only - http://bmjopen.bmj.com/site/about/guidelines.xhtml

**Conclusion**

The cost-effectiveness analysis of the TRIUMPH study showed that there were similar costs and outcomes between the two arms of the trial, indicating that the provision of a manualised and standardised non-pharmacological intervention in general practice can be implemented at neutral cost. This evidence in conjunction with the improvement shown in the effectiveness study, gives support to its implementation within primary care services in the UK.

For peer review only



For peer review only - http://bmjopen.bmj.com/site/about/guidelines.xhtml

## Contributorship statement

Cochrane attests that all listed authors meet authorship criteria and that no others meeting the criteria have been omitted.

*Conception or design:* MC, SN, MDJ, JAL, NC, MF, LM, HH, SM, MJR, ES, JR, LAR and GT assisted with the study design. JW and JF managed the coordination of the study. MM and JT also assisted with the coordination of the study.

*Analysis:* MC and SN conducted the analysis for the economic evaluation.

*Drafting the manuscript:* MC and SN developed the manuscript for the economic evaluation. All authors contributed to the oversight of the study via the TMG, read and commented on manuscript drafts and approved the final manuscript.

*Obtaining funding:* MJD was the Chief Investigator of the TRIUMPH study. The study was funded by National Institute for Health Research (NIHR), Health Technology Assessment (HTA) programme, funding number 16/90/03. The views expressed in this publication are those of the authors and not necessarily those of the NHS, the NIHR or the Department of Health and Social Care. MJR is partially funded by a Post-Doctoral Research Fellowship from the NIHR (PDF-2014-07-013).

## Competing interests

Prof Marcus Drake reports personal fees from Astellas and Pfizer, outside the submitted work. Dr Jonathan Rees is chair of the Primary Care Urology Society which has received non-promotional sponsorship for annual meetings from Ferring, Astellas, Neotract and IMedicare. He has also received speaker fees from Astellas Pharmaceuticals. Prof Hashim Hashim reports personal fees from Medtronic, Astellas, Allergan and Boston Scientific, outside the submitted work. Prof Athene Lane reports receiving funding for the clinical trials unit (CTU) of which she was co-director, and is currently an active member on the NIHR CTU Standing Advisory Committee. Dr Matthew Ridd has been on several NIHR committees including the Systematic Reviews NIHR Cochrane Incentive Awards, HTA General Committee, Evidence Synthesis Programme Grants Committee, NIHR Incentive Awards Committee and is currently on the Evidence Synthesis Programme Advisory Group. Stephanie MacNeill is an active member of the HTA General Committee.

## Funding

This study was funded by the National Institute for Health Research (NIHR) HTA programme, funding number 16/90/03. The funder had no role in the design and conduct of the study; collection, management, analysis, and interpretation of the data; preparation, review, or approval of the manuscript; and decision to submit the manuscript for publication.

Bristol Trials Centre receives National Institute for Health Research CTU Support Funding. This funding has been awarded to support us in developing and supporting NIHR trials. The views



For peer review only - http://bmjopen.bmj.com/site/about/guidelines.xhtml

expressed are those of Bristol Trials Centre and not necessarily those of the NIHR or the Department of Health and Social Care.

## Data sharing statement

All data requests should be submitted to the corresponding author for consideration. Access to anonymised data may be granted following review.

For peer review only

## References

1. National Institute for Health and Care Excellence. Lower urinary tract symptoms in men: management. Clinical Guideline 97 2010 [cited 2021 28th October]. Available from: https://www.nice.org.uk/guidance/cg97
2. Hunter DJ, McKee M, Black NA, Sanderson CF. Health status and quality of life of British men with lower urinary tract symptoms: results from the SF-36. Urology. 1995;45(6):962-71.
3. National Institute for H, Care E. Lower Urinary Tract Symptoms in Men: Management [CG97]. London: NICE; 2010.
4. Brown CT, Yap T, Cromwell DA, Rixon L, Steed L, Mulligan K, et al. Self management for men with lower urinary tract symptoms: randomised controlled trial. Bmj. 2007;334(7583):25.
5. Drake M, Worthington JM, Frost J, Sanderson EJ, Cochrane M, Cotterill N, et al. Treating male lower urinary tract symptoms in primary healthcare using conservative interventions: the TRIUMPH cluster randomised controlled trial. The BMJ. 2023;Accepted/In press.
6. Husereau D, Drummond M, Augustovski F, Bekker-Grob Ed, Briggs AH, Carswell C, et al. Consolidated Health Economic Evaluation Reporting Standards 2022 (CHEERS 2022) statement: updated reporting guidance for health economic evaluations. BMJ. 2022;376:e067975.
7. Frost J, Lane JA, Cotterill N, Fader M, Hackshaw-McGeagh L, Hashim H, et al. TReatIng Urinary symptoms in Men in Primary Healthcare using non-pharmacological and non-surgical interventions (TRIUMPH) compared with usual care: study protocol for a cluster randomised controlled trial. Trials. 2019;20(1):546.
8. NICE. Developing NICE guidelines: the manual. 7 Incorporating economic evaluation2014 [cited 2014 31st October ]. Available from: https://www.nice.org.uk/process/pmg20/chapter/incorporating-economic-evaluation.
9. van Hout B, Janssen MF, Feng Y-S, Kohlmann T, Busschbach J, Golicki D, et al. Interim Scoring for the EQ-5D-5L: Mapping the EQ-5D-5L to EQ-5D-3L Value Sets. Value in Health. 2012;15(5):708-15.
10. Drummond MF, Sculpher MJ, Claxton K, Stoddart GL, Torrance GW. Methods for the Economic Evaluation of Health Care Programmes. Oxford: Oxford: Oxford University Press; 2015.
11. Curtis L, Burns A. Unit Costs of Health and Social Care 2019 Canterbury: Personal Social Services Research Unit; 2020 [cited 2020 17th August]. Available from: https://www.pssru.ac.uk/project-pages/unit-costs/unit-costs-2019/.
12. Pope C, Turnbull J, Jones J, Prichard J, Rowsell A, Halford S. Has the NHS 111 urgent care telephone service been a success? Case study and secondary data analysis in England. BMJ Open. 2017;7(5):e014815.
13. University of Bristol Human Resources. Professorial salary ranges history 2018/19 [cited 2021 2nd March]. Available from: http://www.bristol.ac.uk/hr/salaries/gradem-prof-history.html#a1819.
14. University of Bristol Print Service. Bespoke Printing 2018 [cited 2021 2nd March ]. Available from: http://www.bristol.ac.uk/print-services/bespoke-print-promotional-products-and-signage/.
15. BT. BT Public Sector: Higher education IT solutions 2018/19 [cited 2021 2nd March]. Available from: https://business.bt.com/public-sector/universities/.
16. HM Revenue and Customs. Travel – Mileage and Fuel Rates and Allowances 2019 [cited 2021 2nd March 2021]. Available from: https://www.gov.uk/government/publications/rates-and-allowances-travel-mileage-and-fuel-allowances/travel-mileageand-fuel-rates-and-allowances.
17. National Cost Collection for the NHS. 2018/19 National Cost Collection data: NHS Improvement,; 2020 [cited 2020 17th August]. Available from: https://improvement.nhs.uk/resources/national-cost-collection/.
18. NHS Buisness Services Authority. Prescription Cost Analysis- England 2019: NHS Buisness Services Authority; 2020 [cited 2020 17th August]. Available from: https://www.nhsbsa.nhs.uk/statistical-collections/prescription-cost-analysis/prescription-cost-analysis-england-2019.



For peer review only - http://bmjopen.bmj.com/site/about/guidelines.xhtml

19. NHS Business Services Authority. NHS Drug Tariff 2019 [cited 2021 2nd March]. Available from: https://www.nhsbsa.nhs.uk/pharmacies-gp-practices-and-appliance-contractors/drug-tariff.
20. Curtis L, Burns A. Unit Costs of Health and Social Care 2015 Canterbury: University of Kent; 2015 [cited 2020 17th August]. Available from: https://www.pssru.ac.uk/project-pages/unit-costs/unit-costs-2015/.
21. Cochrane M, Noble SM, Worthington JM, Drake MJ. The TRIUMPH Study: Health Economic Analysis Plan. 2021.
22. Manca A, Hawkins N, Sculpher MJ. Estimating mean QALYs in trial-based cost-effectiveness analysis: the importance of controlling for baseline utility. Health economics. 2005;14(5):487-96.
23. El Alili M, van Dongen JM, Goldfeld KS, Heymans MW, van Tulder MW, Bosmans JE. Taking the Analysis of Trial-Based Economic Evaluations to the Next Level: The Importance of Accounting for Clustering. PharmacoEconomics. 2020;38(11):1247-61.
24. White IR, Royston P, Wood AM. Multiple imputation using chained equations: Issues and guidance for practice. Statistics in medicine. 2011;30(4):377-99.
25. Kwakkenbos L, Imran M, McCall SJ, McCord KA, Fröbert O, Hemkens LG, et al. CONSORT extension for the reporting of randomised controlled trials conducted using cohorts and routinely collected data (CONSORT-ROUTINE): checklist with explanation and elaboration. BMJ. 2021;373:n857.
26. Leurent B, Gomes M, Carpenter JR. Missing data in trial-based cost-effectiveness analysis: An incomplete journey. Health economics. 2018;27(6):1024-40.
27. Thorn JC, Brookes ST, Ridyard C, Riley R, Hughes DA, Wordsworth S, et al. Core Items for a Standardized Resource Use Measure: Expert Delphi Consensus Survey. Value Health. 2018;21(6):640-9.
28. Lewis AL, Young GJ, Selman LE, Rice C, Clement C, Ochieng CA, et al. Urodynamics tests for the diagnosis and management of bladder outlet obstruction in men: the UPSTREAM non-inferiority RCT. 2020;24:42.
29. Watt T, Firth Z, Fisher R, Thorlby R, Kelly E. Use of primary care during the COVID-19 pandemic: Patient-level data analysis of the impact of COVID-19 on primary care activity in England 2020 [cited 2021 30th November 2021]. Available from: https://www.health.org.uk/news-and-comment/charts-and-infographics/use-of-primary-care-during-the-covid-19-pandemic.
30. Gibson W, Harari D, Husk J, Lowe D, Wagg A. A national benchmark for the initial assessment of men with LUTS: data from the 2010 Royal College of Physicians National Audit of Continence Care. World J Urol. 2016;34(7):969-77.
31. Jepson M, Salisbury C, Ridd MJ, Metcalfe C, Garside L, Barnes RK. The 'One in a Million' study: creating a database of UK primary care consultations. British Journal of General Practice. 2017;67(658):e345.

For peer review only

**Figure legend**

Figure 1. Cost-effectiveness acceptability curve from an NHS perspective



For peer review only - http://bmjopen.bmj.com/site/about/guidelines.xhtml

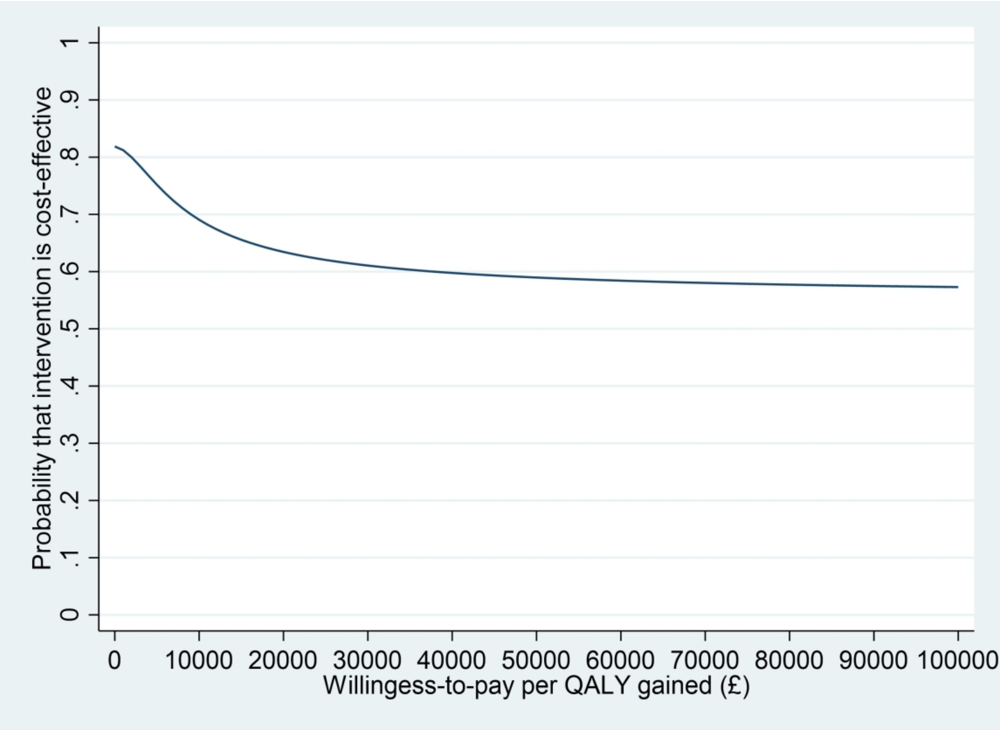


121x88mm (600 x 600 DPI)

Table 1. Output for multilevel model to estimate adjusted mean differences in costs (n=866)

| Total Costs | Coefficient | Bootstrap (SE) | P-value | 95% Confident Intervals |
|---|---|---|---|---|
| Intervention arm | -29.99 | 32.93 | 0.36 | -94.52 to 34.55 |
| Centre | -54.12 | 29.36 | 0.07 | -111.66 to 3.43 |
| GP IT System | -56.73 | 38.20 | 0.14 | -131.59 to 18.14 |
| Practice size | 0.00 | 0.00 | 0.31 | -0.00 to 0.00 |
| IMD | 5.68 | 3.13 | 0.07 | -0.45 to 11.82 |
| Constant | 235.56 | 52.52 | 0.00 | 132.62 to 338.50 |
| ICC | 0.05 | 0.02 | | 0.02 to 0.11 |

SE: Standard Error; IMD: Index of Multiple Deprivation; GP (General Practice) Informatic System; ICC: Intraclass correlation.

Table 2. Output for multilevel model to estimate adjusted mean differences in QALYs (n=866)

| Total QALYs | Coefficient | Bootstrap (SE) | P-value | 95% CI |
|---|---|---|---|---|
| Intervention arm | 0.00 | 0.01 | 0.90 | -0.01 to 0.01 |
| Centre | -0.00 | 0.01 | 0.60 | -0.02 to 0.01 |
| Practice size | 1.96e-07 | 2.75e-07 | 0.47 | -3.42e-07 to 7.35e-07 |
| IMD | -0.00 | 0.00 | 0.32 | -0.00 to 0.00 |
| Baseline utility | 0.81 | 0.018 | 0.00 | 0.78 to 0.85 |
| Constant | 0.16 | 0.019 | 0.00 | 0.13 to 0.20 |
| ICC | 6.48e-35 | 9.76e-19 | | 0 to 1 |

QALYs: Quality Adjusted Life Years; CI: Confidence Intervals; IMD: Indices of Multiple Deprivation; ICC: Intraclass correlation.

For peer review only

Table 3. Additional sensitivity and subgroup analyses

| | | Adjusted*, mean (95% CI)** | | Incremental adjusted mean (95% CI **) | | |
|---|---|---|---|---|---|---|
| Trial arm | n | Costs (£) | QALYs | Costs (£) | QALYs | INMB (£) at £20,000/QALY (95% CI) |
| *Complete Case Analysis with MLM: Adjusting for 12-month pre-consent GP consultation visits* | | | | | | |
| Intervention | 413 | £277.53 (£245.93 to £309.14) | 0.84 (0.83 to 0.84) | -£3.02 (-£49.52 to £44.02) | 0.001 (-0.011 to 0.014) | £21.50 (-£246.93 to £289.03) |
| Usual care | 453 | £280.56 (£244.16 to £316.96) | 0.84 (0.83 to 0.84) | | | |
| *Complete Case Analysis with MLM: Subgroup analysis for participants completing 12-month follow up before 11th March 2020* | | | | | | |
| Intervention | 236 | £255.12 (£133.88 to £376.35) | 0.83 (0.81 to 0.84) | -£33.04 (-£209.94 to £438.39) | -0.001 (-0.027 to 0.010) | £48.01 (-£225.83 to £321.85) |
| Usual care | 169 | £288.16 (£23.20 to £553.11) | 0.84 (0.82 to 0.85) | | | |
| *Complete Case Analysis with MLM: Subgroup analysis for participants completing 12-month follow up from 11th March 2020* | | | | | | |
| Intervention | 177 | £229.31 (£119.77 to £338.85) | 0.86 (0.81 to 0.90) | -£67.77 (-£219.86 to £74.92) | 0.029 (0.002 to 0.133) | £35.54 (-£182.20 to £253.28) |
| Usual care | 284 | £297.07 (£204.54 to £389.61) | 0.83 (0.76 to 0.90) | | | |

*Adjusted for centre, practice-size and area-level deprivation. In addition, costs were adjusted for general practice IT system and QALYs were adjusted for baseline utility.**Bootstrapped bias corrected and accelerated confidence intervals; SUR= Seemingly Unrelated Regression; INMB= Incremental Net Monetary Benefit; MLM= Multilevel model.

For peer review only

# CHEERS 2022 Checklist

| Topic | No. | Item | Location where item is reported |
|---|---|---|---|
| **Title** | | | |
| | 1 | Identify the study as an economic evaluation and specify the interventions being compared. | Title |
| **Abstract** | | | |
| | 2 | Provide a structured summary that highlights context, key methods, results, and alternative analyses. | Abstract |
| **Introduction** | | | |
| **Background and objectives** | 3 | Give the context for the study, the study question, and its practical relevance for decision making in policy or practice. | Introduction |
| **Methods** | | | |
| **Health economic analysis plan** | 4 | Indicate whether a health economic analysis plan was developed and where available. | Methods: Analysis section, First paragraph |
| **Study population** | 5 | Describe characteristics of the study population (such as age range, demographics, socioeconomic, or clinical characteristics). | Methods: Target population and setting section, First paragraph |
| **Setting and location** | 6 | Provide relevant contextual information that may influence findings. | Methods: Target population and setting section, First paragraph |
| **Comparators** | 7 | Describe the interventions or strategies being compared and why chosen. | Methods: Comparators section, First paragraph |
| **Perspective** | 8 | State the perspective(s) adopted by the study and why chosen. | Introduction, second paragraph |
| **Time horizon** | 9 | State the time horizon for the study and why appropriate. | Introduction, second paragraph |
| **Discount rate** | 10 | Report the discount rate(s) and reason chosen. | Methods: Analysis section, First paragraph |

| Topic | No. | Item | Location where item is reported |
|---|---|---|---|
| **Selection of outcomes** | 11 | Describe what outcomes were used as the measure(s) of benefit(s) and harm(s). | Methods: Outcomes section, First paragraph |
| **Measurement of outcomes** | 12 | Describe how outcomes used to capture benefit(s) and harm(s) were measured. | Methods: Outcomes section, First paragraph |
| **Valuation of outcomes** | 13 | Describe the population and methods used to measure and value outcomes. | Methods: Outcomes section, First paragraph |
| **Measurement and valuation of resources and costs** | 14 | Describe how costs were valued. | Methods: Resource use data collection and valuation section |
| **Currency, price date, and conversion** | 15 | Report the dates of the estimated resource quantities and unit costs, plus the currency and year of conversion. | Methods, Table 1 |
| **Rationale and description of model** | 16 | If modelling is used, describe in detail and why used. Report if the model is publicly available and where it can be accessed. | Not applicable |
| **Analytics and assumptions** | 17 | Describe any methods for analysing or statistically transforming data, any extrapolation methods, and approaches for validating any model used. | Methods, Analysis section, first and second paragraph |
| **Characterising heterogeneity** | 18 | Describe any methods used for estimating how the results of the study vary for subgroups. | Methods, Analysis section, last paragraph |
| **Characterising distributional effects** | 19 | Describe how impacts are distributed across different individuals or adjustments made to reflect priority populations. | Not applicable |
| **Characterising uncertainty** | 20 | Describe methods to characterise any sources of uncertainty in the analysis. | Methods: Analysis section |
| **Approach to engagement with patients and others affected by the study** | 21 | Describe any approaches to engage patients or service recipients, the general public, communities, or stakeholders (such as clinicians or payers) in the design of the study. | Methods: Patient and public involvement section, first paragraph |
| **Results** | | | |
| **Study parameters** | 22 | Report all analytic inputs (such as values, ranges, references) including uncertainty or distributional assumptions. | Not applicable as no model |

| Topic | No. | Item | Location where item is reported |
|---|---|---|---|
| **Summary of main results** | 23 | Report the mean values for the main categories of costs and outcomes of interest and summarise them in the most appropriate overall measure. | Results: Tables 2, 3 and 4 |
| **Effect of uncertainty** | 24 | Describe how uncertainty about analytic judgments, inputs, or projections affect findings. Report the effect of choice of discount rate and time horizon, if applicable. | Results: sensitivity analysis section |
| **Effect of engagement with patients and others affected by the study** | 25 | Report on any difference patient/service recipient, general public, community, or stakeholder involvement made to the approach or findings of the study | Methods: Patient and public involvement section, first paragraph |
| **Discussion** | | | |
| **Study findings, limitations, generalisability, and current knowledge** | 26 | Report key findings, limitations, ethical or equity considerations not captured, and how these could affect patients, policy, or practice. | Discussion |
| **Other relevant information** | | | |
| **Source of funding** | 27 | Describe how the study was funded and any role of the funder in the identification, design, conduct, and reporting of the analysis | Role of funding source, page 3 |
| **Conflicts of interest** | 28 | Report authors conflicts of interest according to journal or International Committee of Medical Journal Editors requirements. | Competing interests declaration, page 3 |

*From:* Husereau D, Drummond M, Augustovski F, et al. Consolidated Health Economic Evaluation Reporting Standards 2022 (CHEERS 2022) Explanation and Elaboration: A Report of the ISPOR CHEERS II Good Practices Task Force. Value Health 2022;25. doi:10.1016/j.jval.2021.10.008



For peer review only - http://bmjopen.bmj.com/site/about/guidelines.xhtml
